# The Mesosoma of *Protanilla* (Leptanillinae) and the Groundplan of the Formicidae (Hymenoptera)

**DOI:** 10.1002/jmor.70064

**Published:** 2025-07-22

**Authors:** Lazzat Aibekova, Adrian Richter, Rolf G. Beutel, Thomas van de Kamp, Evan P. Economo, Zachary Griebenow, Brendon E. Boudinot

**Affiliations:** ^1^ Okinawa Institute of Science and Technology (OIST) Okinawa Japan; ^2^ Division Messel Research & Mammalogy, Evolution of Sensory Systems Section Senckenberg Research Institute and Natural History Museum Frankfurt am Main Germany; ^3^ Institut für Zoologie und Evolutionsforschung FSU Jena Jena Germany; ^4^ Division of Terrestrial Zoology, Section Entomology II Senckenberg Research Institute and Natural History Museum Frankfurt am Main Germany; ^5^ Institute for Photon Science and Synchrotron Radiation (IPS) Karlsruhe Institute of Technology (KIT) Eggenstein‐Leopoldshafen Germany; ^6^ Laboratory for Applications of Synchrotron Radiation (LAS) Karlsruhe Institute of Technology (KIT) Karlsruhe Germany; ^7^ Department of Entomology University of Maryland College Park Maryland USA; ^8^ College of Agricultural Sciences, Agricultural Biology Colorado State University Fort Collins Colorado USA; ^9^ Systematic Entomology Laboratory, United States Department of Agriculture Agricultural Research Service c/o National Museum of Natural History Washington District of Columbia USA

**Keywords:** 3D morphometrics, anatomy, character evolution, micro‐CT, thorax

## Abstract

The study of ant morphology is advancing through parallel insights provided by phylogenomics—which provides a statistically robust basis for comparison and evolutionary inference—and phenomics via the application of microcomputed tomography (µ‐CT) for the efficient and precise documentation of anatomy. The information provided by µ‐CT is complex and rich, allowing for the quantification of geometry and biomechanically relevant variables, as well as comparative morphology via 3D rendering. Recently, the complete musculature of the thorax, propodeum, and legs was documented for the first time in an ant (*Formica rufa* L.). Here, we provide a detailed comparison of those findings for *Protanilla lini* Terayama, 2009 (Leptanillinae), representing the Leptanillomorpha, a clade putatively sister to all other living ants. Using µ‐CT, computer‐based reconstruction, and scanning electron microscopy (SEM), we observe a novel series of morphological features that are plausibly part of the groundplan of the Formicidae. For several of these features, we provide new anatomical concepts and terms, with special discussion of the pronotum, promesothoracic articulation, and metapleural gland region. We also observe characters that are likely correlated with specialized subterranean habits, including the increased flexibility of the promesothoracic articulation, the slender shape of the mesosoma, a simplified vestiture of short setae, and depigmentation. Mesosomal skeletomusculature in *P. lini* appears to be plesiomorphic relative to other Leptanillomorpha, resembling the putative ancestral condition for the Formicidae. An exception lies in the lack of cervical muscle (Idlm1) in *Protanilla* that is present in almost all other insects for which this character has been sampled. With this study of *P. lini*, we are one step closer to realizing the complete set of defining features and variation of the ant mesosoma.

## Graphical abstract

Phenomic investigation of the mesosoma of *Protanilla lini* reveals groundplan features of the ants and raises new homology questions for future investigation, both within Formicidae and across Aculeata. Special highlights include refined definition of the pronotum, complex discoveries of the promesothoracic articulation, and a new perspective on and terminology for the metapleural gland region.

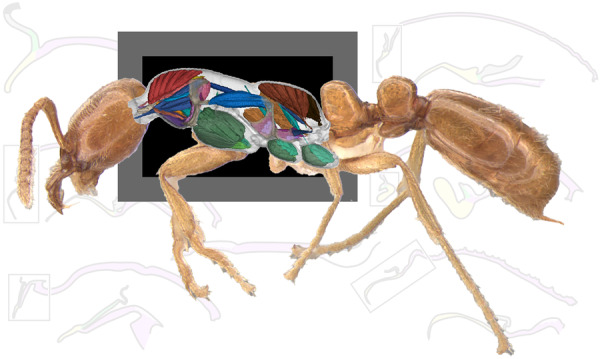

## 1 Introduction

Ants (Formicidae) are a conspicuous and ecologically dominant group that has been intensively investigated (see, e.g., Wilson 1971; Hölldobler and Wilson 1990; Bolton 1994, 2003; Lach et al. 2009; Ward 2014; Branstetter et al. 2017). Nevertheless, many aspects of ant anatomy were neglected or overlooked for a long time, including the mesosoma, which is crucial for carrying prey, brood, and other objects, and for locomotion (e.g., Keller et al. 2014; Liu et al. 2019; Peeters et al. 2020; Aibekova et al. 2022). Early studies on the mesosoma of Formicidae were published more than 100 years ago (e.g., Lubbock 1881; Nasonov 1889; Janet 1897, 1898; Emery 1900). They cover only four species and are of somewhat limited use due to simple techniques of morphological investigation and documentation. More recent studies treated *Formica* (Markl 1966; Aibekova et al. 2022), *Camponotus* (Saini et al. 1982), and *Myrmecia* (Liu et al. 2019). The studies of Liu et al. (2019) and Aibekova et al. (2022) were the first anatomical treatment of ant mesosomata based on microcomputed tomography (µ‐CT) and computer‐based 3D reconstruction.

The study of ant morphology is advancing through parallel insights provided by phylogenomics, which provides a more concrete basis for comparison and evolutionary inference, and particularly via the application of µ‐CT for anatomical documentation. Recent works using µ‐CT have transformed our knowledge of the head (e.g., Richter et al. 2021, 2022, 2023), metasoma (Lieberman et al. 2022), male genitalia (Griebenow et al. 2023; Boudinot et al. 2024), and the mesosoma (Liu et al. 2019; Aibekova et al. 2022, 2023). Here, we revisit the mesosoma by sampling *Protanilla lini*, a species of the phylogenetically key subfamily Leptanillinae. The leptanillines are a clade of subterranean ants comprised of three genera and, at present, 82 species (Griebenow 2024; Bolton 2025). Together with the mysterious and elusive *Martialis* Rabeling et al. 2008, Leptanillinae is likely the sister group of the remaining crown Formicidae (e.g., Borowiec et al. 2019, 2025; Romiguier et al. 2022). While there are clear external patterns of adaptation to their subterranean lifestyle (e.g., Boudinot 2015; Boudinot et al. 2025), virtually nothing is known about the skeletomuscular anatomy of worker Leptanillinae, except for a pair of recent studies on the head of *P. lini* and *Leptanilla* (Richter et al. 2021; Griebenow et al. 2025).

Similar to the earlier studies of Liu et al. (2019) and Aibekova et al. (2022), we employed µ‐CT scanning, computer‐based 3D reconstructions, and SEM images to document external and internal mesosomal structures of *P. lini*, emphasizing the skeletomuscular system. The observed structural features are discussed in a phylogenetic, functional, and evolutionary context. We attempt to identify preserved groundplan character states of the crown and total clades of Formicidae (i.e., excluding or including the stem group). Features that are likely linked with hypogaeic habits and miniaturization are addressed. Finally, we tentatively compare the mesosomal condition of *P. lini* to that in other Leptanillomorpha *sensu* Richter et al. (2021), so far as is possible based on existing literature. Coupled with the study of Richter et al. (2021) on cephalic anatomy in *P. lini*, this study contributes to the understanding of the morphology and evolution of a phylogenetically crucial subfamily. We find that critical examination of *Protanilla lini* from the phenomic perspective considerably enriches our understanding of the groundplan of Formicidae.

## 2 Material and Methods

### 2.1 Material

Two specimens of *P. lini* Terayama, 2009 were used in this study. The one used for SEM was collected in Yuchih Township, Nantou County, Taiwan, on 7.XII.2015 by Po‐Cheng Hsu using hand‐collection (see also Hsu et al. 2017) and preserved in 70% ethanol. This entirely depigmented and fragile specimen with the unique identifier CASENT0790210 was also used by Richter et al. (2021) to study the head. The 3D renderings presented here were based on a worker of *P. lini* stored in ethanol (collection code OK01772, Japan, Okinawa, Uruma. Yacho‐no‐mori, 26.37581°, 127.87071°, 30 m, SLAM trap, 15‐29.vi.2016, OKEON) with the unique specimen identifier CASENT0741315.

### 2.2 Conventional µ‐CT Scanning and 3D‐Reconstruction

The specimen CASENT0741315 was stained in iodine. The staining was performed as part of a rolling batch, with at least 1 week of staining per specimen. The scanning parameters at a Zeiss Xradia 510 Versa 3D X‐ray microscope operated with the Zeiss Scout‐and‐Scan Control System software (version 14.0.14829.38124) were 40 kV and 3 W beam strength, 3 s exposure time, under 4X objective and 2001 projections, which resulted in 1.23 μm voxel size. Postprocessing was done in Amira 2019.2 software (Visage Imaging GmbH, Berlin, Germany). The 3D renderings were done on VGStudio 2022.1 (Volume Graphics GmbH, Heidelberg, Germany).

### 2.3 Synchrotron‐Radiation µ‐CT

Synchrotron microtomography (SR‐µ‐CT) was performed at the imaging cluster of the KIT Light Source using a parallel polychromatic X‐ray beam produced by a 1.5 T bending magnet. The beam was spectrally filtered by 0.5 mm aluminum with a spectrum peak at about 15 keV. We employed a fast indirect detector system, consisting of a scintillator (2x: 200 µm LuAG: Ce; 5x: 25 µm LSO: Tb; 10x: 13 µm LSO: Tb [Cecilia et al. 2011]), and a diffraction limited optical microscope (Optique Peter, Lentilly, France; Douissard et al. 2012) coupled with a 12 bit pco.dimax high speed camera with 2016 × 2016 pixels (dos dos Santos Rolo et al. 2014). The specimens were scanned in 95% ethanol. We took 3000 projections at 70 fps at 2x, 5x, or 10x optical magnifications (Table 1), resulting in an effective pixel sizes of 6.11, 2.44, or 1.22 µm, respectively. Since some of the samples were too large to fit in the vertical field of view, they were scanned in multiple height steps. The control system concert (Vogelgesang et al. 2016) was used for automated data acquisition and online reconstruction of tomographic slices for data quality assurance. The final tomographic 3D reconstructions were performed with tofu (Faragó et al. 2022) and included phase retrieval (Paganin et al. 2002), ring removal, 8‐bit conversion, and blending of phase and absorption 3D reconstructions to increase contrast between the background and homogeneous regions, while at the same time highlighting the edges.

**Table 1:** Synchrotron microtomographic scans used in the present study.

Identification and source information	Specimen, Scan, Mag.
Outgroups	
1. Ampulicidae: *Ampulex* nr. *moebii*	SMFENT0001921, BEB‐KIT033, 5x
Source: Ghana, Western Region: Nini Suhien NP; Ankasa Game Reserve; 5.248° 2.648°, 80 m; 2024‐iv‐24; FFP14GHA53; leg. Hauser, M. & Gaimari, S.	
2. Vespidae: Eumeninae, gen. et sp. indet.	SMFHYM0005665, BEB‐KIT311, 2x
Source: Same data as in Meira et al. (2024).	
3. Scoliidae: Scoliinae, gen. et sp. indet.	USNMENT01900110, BEB‐KIT049, 5x
Source: Madagascar, Antsiranana: Montagne d'Ambre NP; −12.541° 49.168°; 1190 m; 2017‐ii‐13; FFP17MAD52; leg. Borkent, C.	
4. Apidae: *Thyreus quinquifasciatus*	SMFHYM0005662, BEB‐KIT311, 2x
Source: Meira et al. (2024)	
5. Formicidae: Amblyoponinae: *Mystrium* sp.	CASENT0842705, BEB‐KIT221, 5x
Ghana, Central: nr. Abrato, Kakum National Park; 5.35278° −1.39167°; 185 m; 2014‐iv‐19–v‐01; Malaise trap, ex. 2 m MT (14‐8); leg. Gaimari, S. & Hauser, M.	
6. Formicidae: Ectatomminae: *Rhytidoponera metallica*	USNMENT01900478, BEB‐KIT197, 5x
Source: Australia, Queensland: Mt. Windsor Tableland; 16.28333° 145.06667°; 1000 m; 1980‐vi‐04; PSW04407; leg. Ward, P. S.	
7. Formicidae: Amblyoponinae: *Amblyopone australis*	CASENT0753222, BEB‐KIT2019‐08b, 5x
Source: Lieberman et al. (2022).	
6. Formicidae: Leptanillinae: *Leptanilla* cf. *charonea*	Batch specimen identifier: BEB‐KIT2019‐07, 10x
Unknown original collecting conditions due to corruption of metadata file during a hardware failure event in 2020; specimen collected by J. M. Gómez‐Duran in Spain.	

### 2.4 Scanning Electron Microscopy

The specimen CASENT0790210 was previously used for µCT scans of the head (Richter et al. 2021), after which it was removed from its pipette tip sample holder, transferred to 100% acetone and dried at the critical point in liquid CO_2_ with an Emitech K 850 Critical Point Dryer (Sample Preparation Division, Quorum Technologies Ltd., Ashford, England). The specimen was then glued on the tip of a minute needle laterally on its metasoma. It was subsequently sputter coated with gold using an Emitech K 500 (Sample Preparation Division, Quorum Technologies Ltd., Ashford, England). The rotatable specimen holder (Pohl 2010) allowed taking SEM micrographs of the mesosoma and legs from various angles in a Philips ESEM XL30 (Philips, Amsterdam, Netherlands) equipped with Scandium FIVE software (Olympus, Münster, Germany).

### 2.5 Terminology

The majority of mesosomal terms used herein are from Aibekova et al. (2022), with musculature terminology derived from Friedrich and Beutel (2008) and Beutel et al. (2014). We revise the prosternal terminology based on Meira et al. (2024) and ongoing investigation. Tarsal structures are referred to and labeled after Beutel et al. (2020). As in Aibekova et al. (2022) we aim at compatibility with the Hymenoptera Anatomy Ontology as far as possible (Yoder et al. 2010: HAO) (see Aibekova et al. 2022). Where new anatomical concepts were necessary, we have marked these with asterisks (*). Specifically, we introduce a new term here for the often elevated mesonotal contact surface to the pronotum of worker ants; the mesonotal glissella*, after the Proto‐Germanic *glidan* “to glide.” We have corrected the application of some terms, notably in reference to the “cervical prominences” (Aibekova et al. 2022), which we now recognize are actually “cervical apodemes” (Vilhelmsen 2000a).

## 3 Results

### 3.1 General Appearance

The total length for the body of this slender species is ca. 2.5 mm. The sides of the mesosoma are subparallel in dorsal view, narrower than the posterior region of the distinctly prognathous head and the metasoma at its greatest width. The cuticle is mostly smooth and shiny and of a light brown to yellowish coloration. Most areas of the surface are covered with a moderately dense and fairly uniform vestiture of setae of ca. 80 µm length (type 1). Some areas of the cuticle are glabrous, for instance the anterolateral area of the propodeum and the posterior surfaces of the coxae.

### 3.2 Mesosomal Skeleton

The mesosoma comprises the three thoracic segments and abdominal tergum I (propodeum) (e.g., Snodgrass 1910; Tulloch 1935; Vilhelmsen 2000a; Aibekova et al. 2022); it is elongate, c. 0.78 mm (Weber's length) and slightly more than 4x as long as the maximum width in the middle region of the pronotum. The *pronotum* freely articulates with the *mesonotum*, while the meso‐ and metathorax are completely fused to one another and to the propodeum, thus forming the ***oligopropodosoma*** * (=mesometathoracicopropodeal complex; in contrast to the holopropodosoma* = completely fused promesometathoracicopropodeal complex as observed in, e.g., *Tatuidris*, Proceratiinae, Myrmicinae, and Mutillidae, and the propodosoma* = metathoracicopropodeal complex, as observed in Apocrita generally).

### 3.3 Prothorax

**Figure 1:**
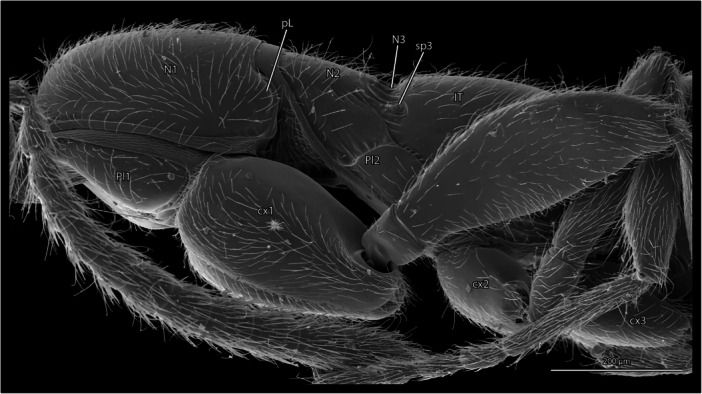
*Protanilla lini*, mesosoma in lateral view. **cx1‐3 **= pro‐, meso‐, and metacoxa; **N1, 2 **= pro‐, mesonotum, N3 = metanotal sulcus, **pL **= pronotal lobe, **Pl1–2 **= propleuron, mesopleural region, **sp3 **= metathoracic spiracle.

**Figure 2:**
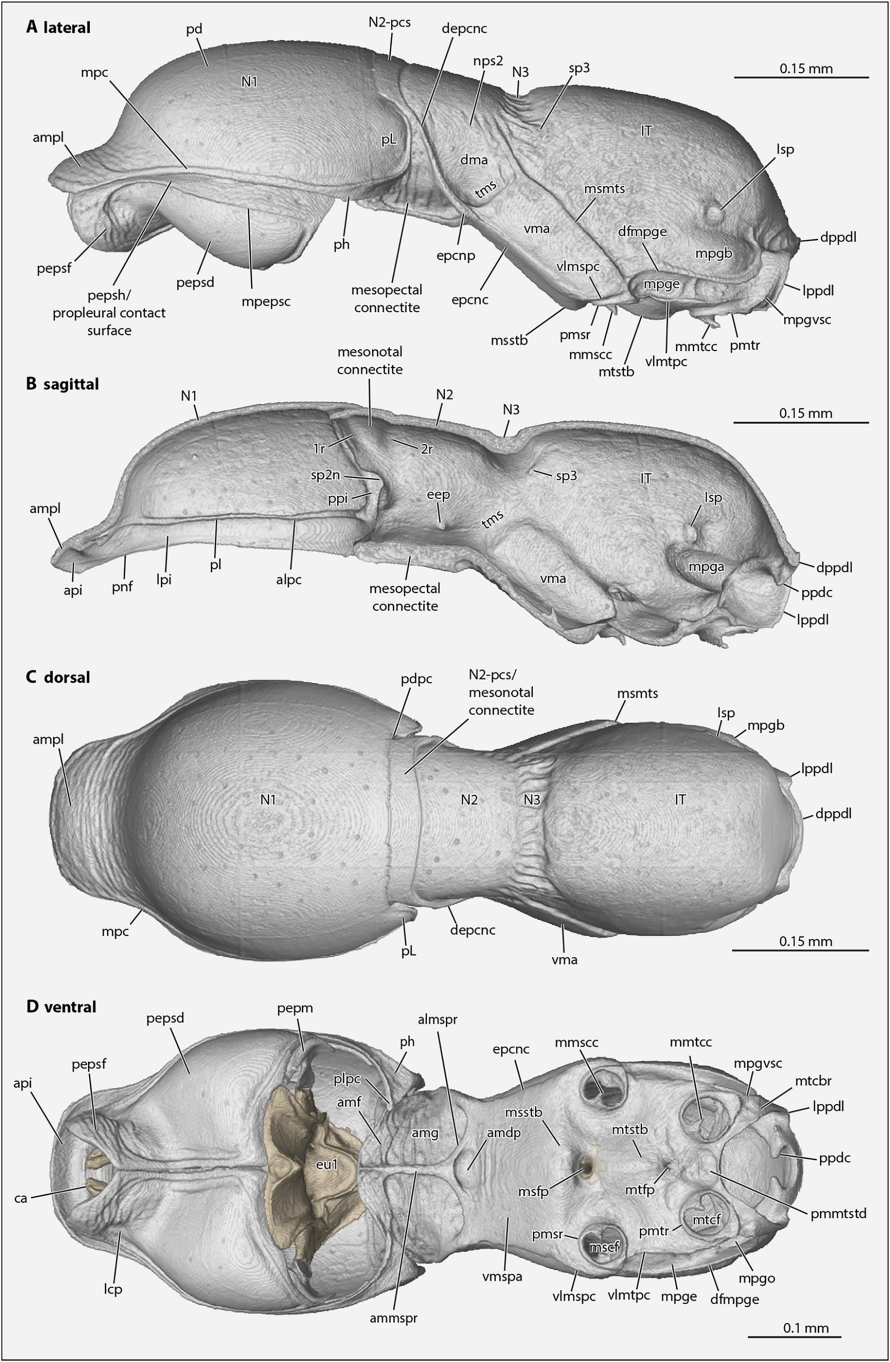
Mesosomal exoskeleton of *Protanilla lini* with legs removed. (A) lateral view; (B) sagittal view with ventral part of prothorax removed; (C) dorsal view; (D) ventral view. **almspr **= anterolateral mesopectal ridge, ammspr = anteromedial mesopectal ridge, **alpc **= anterolateral pronotal corner, **amdp **= anterior mesodiscrimenal pit, **amf **= anterior mesopectal flange, **amg **= anterior mesopectal groove, **ampl **= anteromedial pronotal lobe, **api **= anterior pronotal inflection, **ca **= cervical apodeme, **depcnc **= dorsal epicnemial carina, **dfmpge **= dorsal flange of the metapleural gland evaporatorium, **dma **= dorsal mesopleural area, **dppdl **= dorsal propodeal lobe, **eep **= evagination of the epicnemial process, **epcnc **= epicnemial carina, **epcnp **= epicnemial process, **eu1 **= eusternum, **lcp **= laterocervical pit, **lpi **= lateral pronotal inflection, **lppdl **= lateral propodeal lobe, **Isp **= propodeal spiracle, **IT **= propodeum, **mmscc **= medial mesocoxal condyle, **mmtcc **= medial metacoxal condyle, **mpc **= marginal pronotal carina, **mpepc **= marginal proepisternal carina, **mpgb **= metapleural gland bulla, **mpgba **= metapleural gland ampulla, **mscf **= mesocoxal foramen, **msfp **= mesofurcal pit, **msmts **= mesometapleural suture, **msstb **= mesosternal bulge, **mtcbr **= metacoxal bridge, **mtcf **= metacoxal foramen, **mtfp **= metafurcal pit, **mtstb **= metasternal bulge, **N1, 2 **= pro‐, mesonotum, **N2‐pcs **= pronotal contact surface of mesonotum, **N3 **= metanotal groove, **nps2 **= mesonotopleural suture, **pd **= pronotal disc, **pdpc **= posterodorsal pronotal corner, **pepm **= proepimeron, **pepsd **= proepisternal disc, **pepsf **= proepisternal flange, **pepsh **= proepisternal hypomeron, **ph **= pronotal hypomeron, **pl **= pronotal ledge, **pL **= pronotal lobe, **plpc **= posterolateral pronotal corner, **pmmtstd **= posteromedian metasternal depression, **pmsr **= periarticular mesocoxal rim, **pmtr **= periarticular metacoxal rim, **pnf **= pronotal flange, **ppdc **= propodal condyle, **ppi **= posterior pronotal inflection, **sp2n **= mesospiracular notch, **sp3;**= metathoracic spiracle, **tms;**= transverse mesopleural sulcus, **vlmspc **= ventrolateral mesopectal carina, **vlmtpc **= ventrolateral metapectal carina, **vma **= ventral mesopleural area, **vmspa **= ventral mesopectal area.

**Figure 3:**
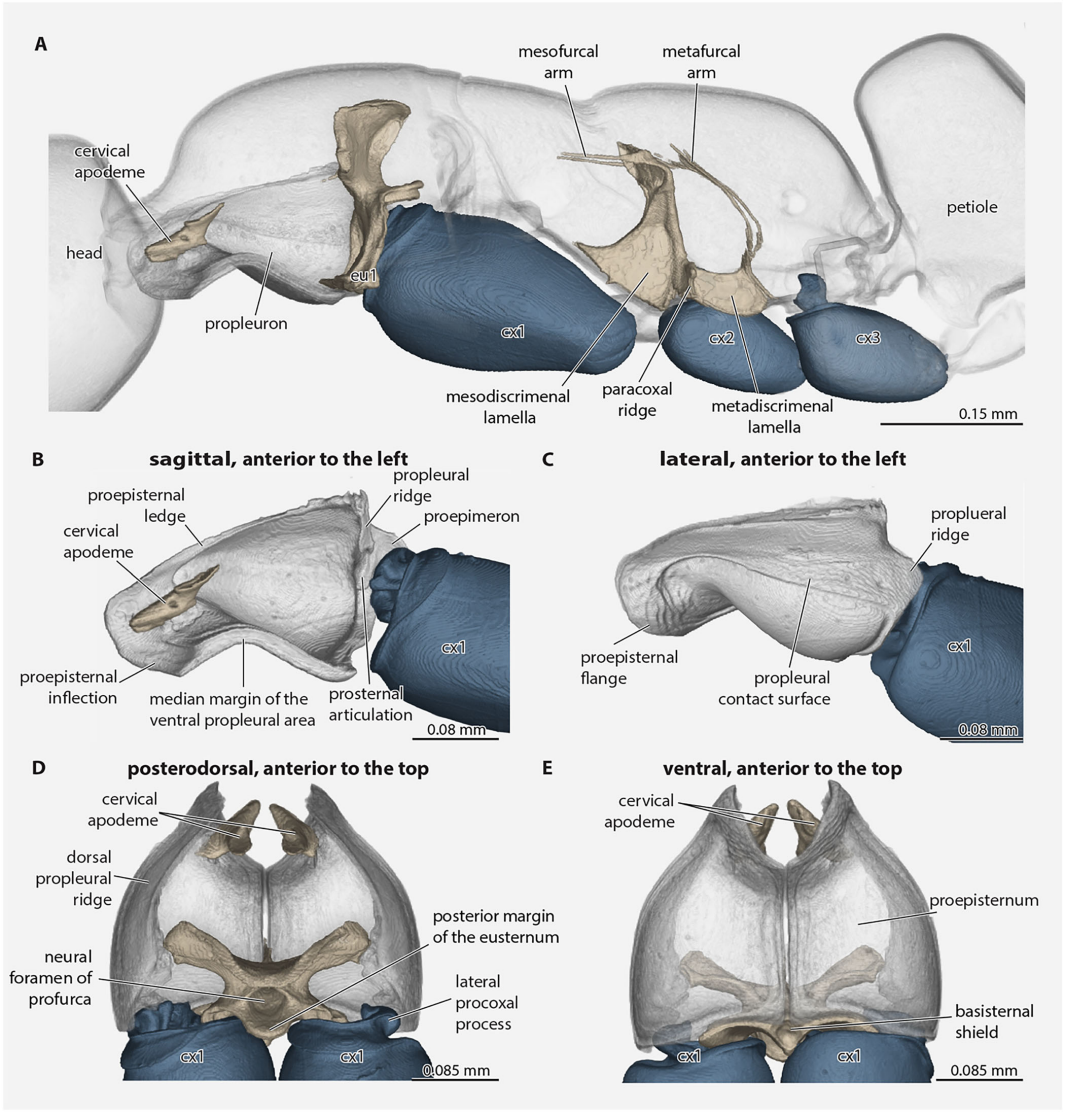
*Protanilla lini*. (A) posterior head capsule, mesosoma with coxae, and petiole (major part), with cervical apodeme and furcae. (B) Mesal view of propleuron and proximal procoxa. (C) Mesal view of propleuron and proximal procoxa. (D) Dorsal view of dorsally open prothorax, with cervical apodeme, profurca, and proximal procoxa. (E) Ventral view of prothorax (rendered semitransparent), with cervical apodeme, profurca and proximal procoxa. **cx1–3 **= pro‐, meso‐, metacoxa, **eu1 **= eusternum.

The **prothorax** is about 1/3 as long as the oligopropodosoma (Figures 1, 2, 3); it comprises the strongly sclerotized dorsal *pronotum* and the ventral *propectus* (=pleurosternal complex), which consists of the propleural halves and the prosternum; this structural complex articulates with the head capsule anteriorly and provides anchorage for the procoxae posteroventrally (Figure 3) (Snodgrass 1942; Aibekova et al. 2022). The **cranial articulation** comprises the *pronotal neck*, the *propleural neck*, and the *cervical apodemes* (Figure 3A), which are fused to the propleurae; this complex articulation fits into the concave *postoccipital region* of the head (HAO: occiput) above and below the narrow *foramen occipitale*. The ***pronotal*** and ***propleural necks*** are the anterior lobate extensions that include both the sclerotic *anterior pronotal* and *propleural lobes*, as well as their associated intersegmental membrane between the head and thorax.

The **pronotum** (**N1**, Figures 1 and 2A,C) is shield‐like and well‐sclerotized; in profile (=lateral) view it is unevenly sinuate, with the short neck region curving to the much larger, dome‐shaped muscle‐bearing portion of the sclerite; its lateral surfaces curve downwards, almost in a saddle‐like manner; it is distinctly narrowed anteriorly in lateral, dorsal, and ventral view (Figure 2A,C,D), forming the ***anteromedial pronotal lobe***, which corresponds to the “pronotal flange” of Aibekova et al. (2022), which comprises the anteriormost portions of both the pronotal flange and disc, and which is ca. 0.1 mm wide at its anterior edge; in lateral view from its anterior margin, the pronotum strongly widens dorsally in an even curve, reaching the maximum width of ca. 0.35 mm at about its midlength; also in lateral view, its ventral margin is shallowly sinuate, without a distinct anterolateral corner; in dorsal or ventral view (Figure 2C,D) its lateral margins are nearly parallel in the posterior portion for a short length before curving posteromedially to its posterior margins; in lateral view (Figure 2A), its lateral margins evenly curve into the posterior margin dorsomedially, forming the distinct ***pronotal lobes*** (**pL**), which conceal the mesothoracic spiracle (**sp2**) and which end dorsally at above the midheight of the sclerite, where the posterior pronotal margin becomes transverse. The margins of the pronotum are continuously expanded along its sides away from the lumen of the prothorax as the ***pronotal flange*** * as seen in sagittal view (Figure 2B), with this flange divisible into the ***anterior, lateral***, and ***posterior regions of the pronotal flange*** *, for which the anterior region corresponds to the “pronotal rim” of Aibekova et al. (2022); these regions of the pronotal flange are distinguished by the ***anterolateral*** and ***posterolateral corners of the pronotum*** *, of which the anterolateral corner is vestigial and scarcely discernible. The internal yet not lumenal surface of the pronotal flange is the ***pronotal inflection*** *, which is divisible into the ***anterior***, ***lateral***, and ***posterior regions of the pronotal inflection*** * by the ***anterolateral***, ***posterolateral*** (plpc), and posterodorsal corners of the pronotum* (Figure 2B–D); the anterior and lateral regions of the inflection contact the propleurae, while the posterior region overlaps the mesonotum anteriorly. Externally, the pronotum is margined along its anterior, lateral, and posterolateral edges by the ***marginal pronotal carina*** * (or “bead”), which continues from near the dorsalmost point of the pronotal lobe completely across the pronotum; this carina delimits the medial ***pronotal disc*** * from the marginal ***pronotal hypomeron*** *, which is a distinctly raised region of marginal cuticle that is wide along the pronotal neck, narrows to the lateral margins, where it is wider than 12 µm and expands posteriorly to the posterolateral pronotal corner—forming the *posteromedial pronotal expansion*—where it also curves ventromediad around the procoxal bases before narrowing along the posterior margin of the pronotal lobe; this portion of the hypomeron does not meet posterad the procoxae, thus a postcoxal bridge is not formed and the procoxal cavities are open posteriorly (Figure 2D); it displays an indistinct surface pattern of longitudinal riffles, and it does not strictly mirror the internal margin of the prothoracic lumen. Internally, the pronotum bears the ***pronotal ledge*** *, which is a distinct ridge that marks the boundary between the pronotal lumen and flange.

**Figure 4:**
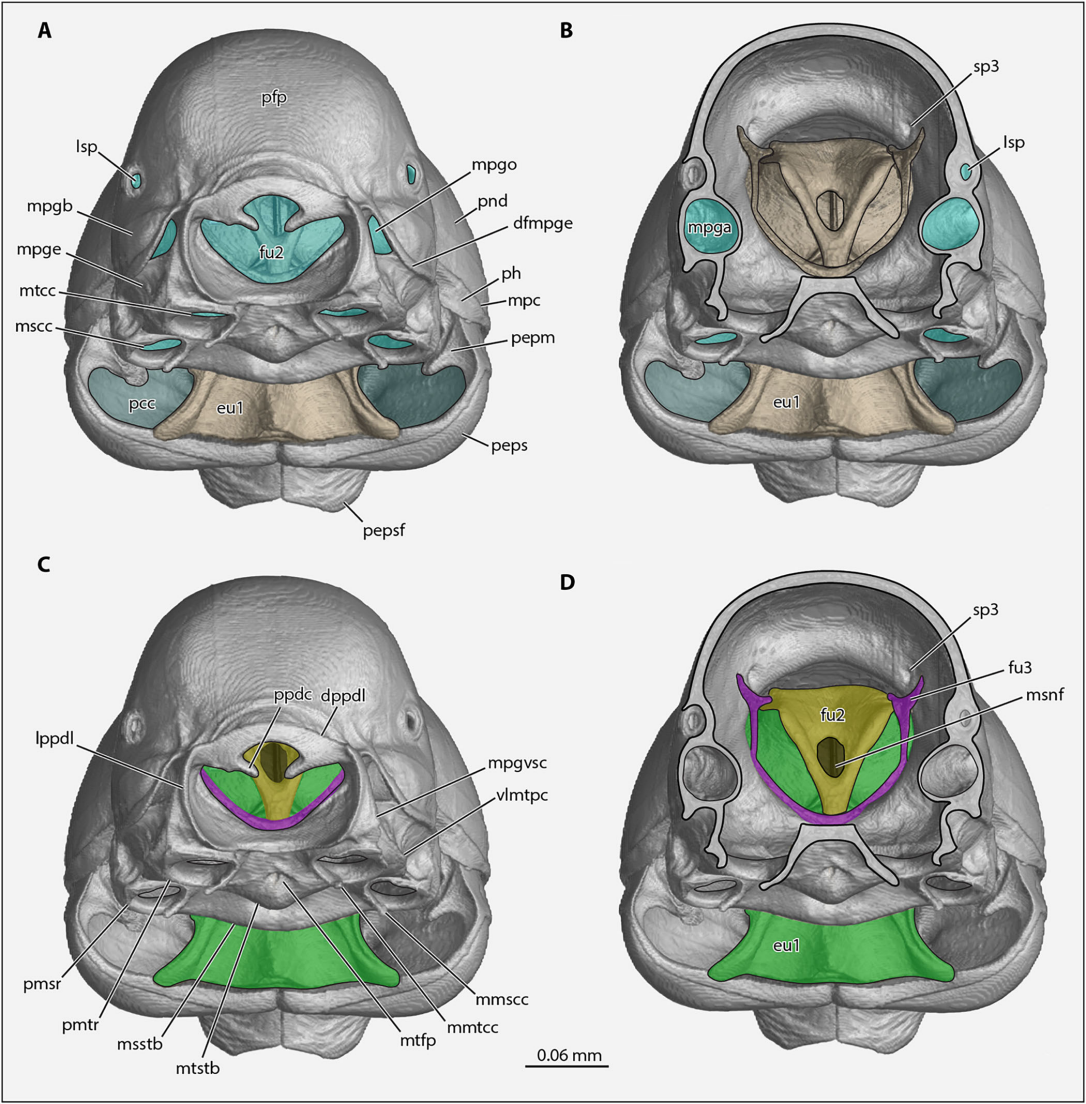
*Protanilla lini*, mesosoma in ventrally oblique posterior view. (A and C) Mesosoma whole. (B and D) Mesosoma with oligopropodosoma transversely sectioned through the propodeal spiracles and metacoxal foramina. **dfmpge **= dorsal flange of the metapleural gland evaporatorium, **dppdl **= dorsal propodeal lobe, **fu2 **= mesofurca, **lppdl **= lateral propodeal lobe, **Isp **= propodeal spiracel, **mmscc **= medial mesocoxal carina, **mmtcc **= medial metacoxal condyle, **mpc **= marginal pronotal carina, **mpgb **= metapleural gland bulla, **mpgo **= metapleural gland orifice, **mpgvsc **= metapleural gland ventral subtending carina, **msnf **= neural foramen of the mesofurca, **mtfp **= metafurcal pit, **pepm **= proepimeron, **peps **= proepisternum, **pfp **= propodeum, **ph **= pronotal hypomeron, **pmsr **= periarticular mesocoxal rim, **pmtr **= periarticular metacoxal rim, **pnd **= pronotal disc, **ppdc **= propodeal condyle, **vlmtpc **= ventrolateral metapectal carina.

The **propleurae** are freely articulating paired halves (Figures 2D, 3D,E, and 4); they are situated between the head, procoxae and prosternal complex; they are strongly developed and well‐sclerotized; they are about 3/4 as long as the entire prothorax; they are largely congruent with the outline of the pronotum in dorsal view; and each propleuron is divided into the anterior *proepisternum* and posterior *proepimeron* by the ***propleural ridge***, which bears the ***prosternal*** and ***procoxal articulations*** at about midheight (Figure 3B,C). The ***proepisterna*** together appear bell‐shaped in ventral view, as they narrow anteromedially to the *propleural neck*, which encloses the anteromedian cervical membrane; each proepisternum is pointed anteriorly as seen in ventral view, with the *lateral proepisternal margin* widely and sinuously curving to the propleural ridge posteriorly, and the *anteromedial proepisternal margin* narrowly and sinuously curving to the *posteromedial proepisternal margin*, which is linear and contacts the opposite proepisternum along its length; each proepisternum is also margined by the ***marginal proepisternal carina*** *, which delimits the medial proepisternal disc* from the marginal proepisternal hypomeron*. The ***proepisternal disc*** * is concave anteriorly, bears the *laterocervical pit* (Figure 2D), and is strongly bulging posteriorly, with this bulge grossly expanded below the pronotum as seen in lateral view. The ***proepisternal hypomeron*** * completely encircles the proepisternal disc; it is broad anteriorly in the region of the propleural neck and narrows laterally where it also bends dorsomedially, forming the lateral *proepisternal contact surface* before strongly narrowing along the posterior proepisternal margin, along which it continues as a narrow region through the medial proepisternal margin to the pronotal neck; it also has a distinct longitudinal pattern of flat scale‐like structures and a pattern of transverse wrinkles. The anterior and anterolateral margins of the proepisternum are expanded, forming the ***proepisternal flange*** *, which corresponds internally to the ***proepisternal inflection*** * (Figure 3B); this inflection is continuous with the ***proepisternal ledge*** (=Snodgrass 1942: horizontal apodeme of episternum; Markl 1966: Proepisternalschaufel; Vilhelmsen 2000b: propleural ledge; HAO: dorsal propleural ridge), which extends into the prothoracic lumen mesally, bears muscular origins, and is continuous posteriorly with the propleural ridge. Internally, each proepisternum bears the strongly developed ***cervical apodeme*** (ca, Figures 2D and 3D,E) (Markl 1966: Querapodem; mislabeled as “cervical prominence”: Aibekova et al. 2022; Vilhelmsen 2000b: cervical sclerites always fused with propleura in Hymenoptera); this strongly sclerotized structure bears a large, apically rounded anterior process which almost reaches the sagittal plane, and a smaller rounded posterior process; the anterior process is part of the articulation with the cephalic postoccipital region. The ***proepimeron*** is developed as a lamella posterad the propleural ridge (Figure 3B); it broadens from the medial propleural margin to its greatest width posterad the prosternal and procoxal articulations before strongly narrowing and becoming virtually absent along the dorsalmost portion of the propleural ridge.

**Figure 5:**
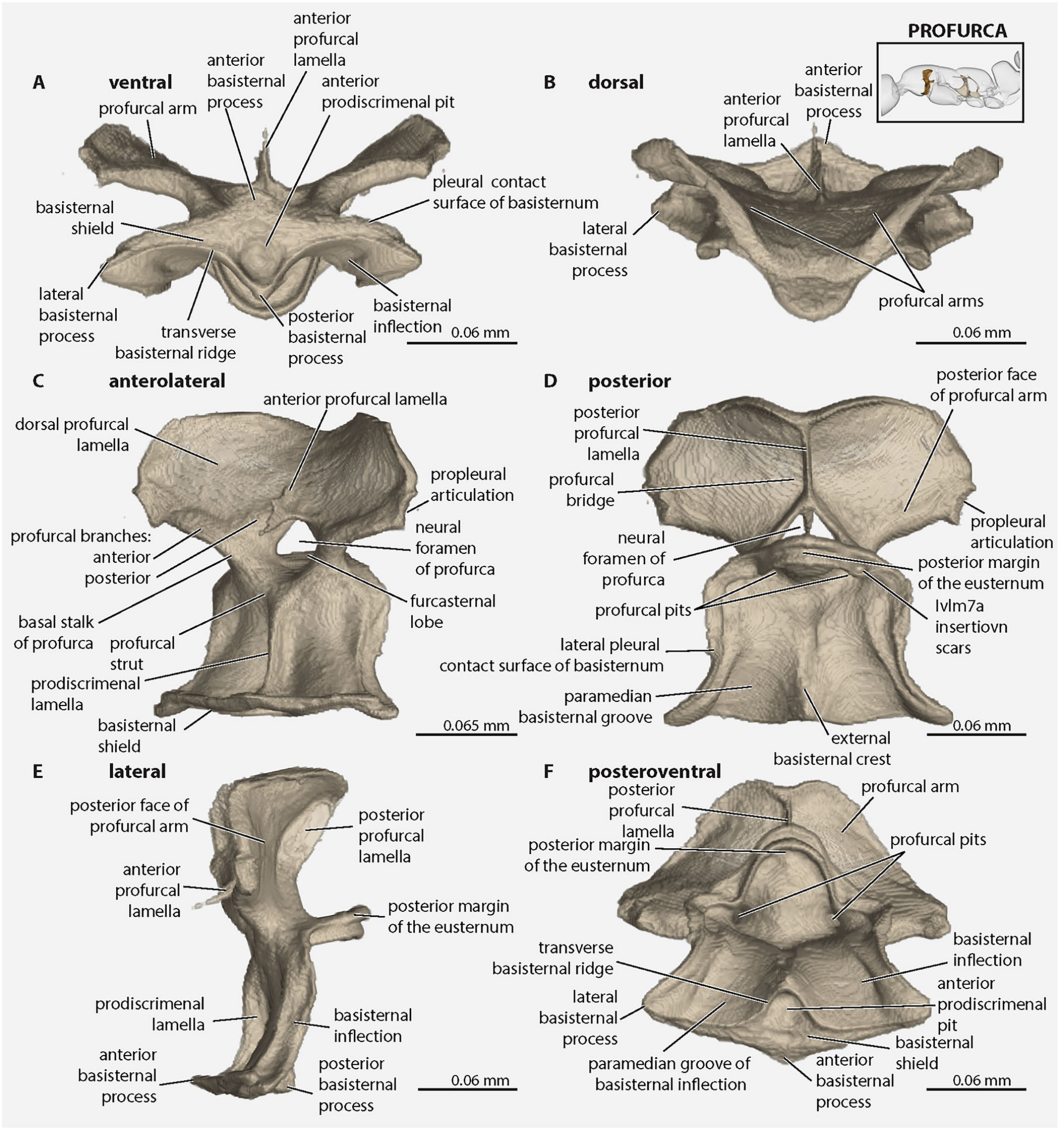
*Protanilla lini*, prosternum. (A) Ventral view; (B) dorsal view; (C) anterolateral view; (D) posterior view; (E) lateral view; (F) posteroventral view.

The **prosternum** consists solely of the *eusternum* (Figures 2D and 5D) as the *prospinasternum* is absent. The ***eusternum*** is situated posteromediad the propleurae and anterad the procoxae; it is divided into the anterior *basisternal region* and the posterior *furcasternal region* by an imaginary line drawn between the *profurcal pits*, which correspond to invaginations that form the *profurcal arms* (Figure 3A); internally, it bears the short ***prodiscrimenal lamella***, which ends slightly anterad the bases of the profurcal arms, and which bears along its dorsal margin the very weakly defined ***profurcal struts*** (Figure 5). The ***basisternal region*** is further divided into the anterior *basisternal shield* and posterior *basisternal inflection* by the *transverse basisternal ridge*, which is sharply margined in ventral view (Figure 5A); the shield and inflection are offset at roughly 90° in lateral view (Figure 5E). The ***basisternal shield*** is the only part of the eusternum that is visible externally; it is diamond‐shaped (quadrangular) being about 4x as wide lateromedially as long anteroposteriorly, and with the distinct yet oblique ***anterior basisternal process***, narrow ***lateral basisternal processes***, and the acutely triangular ***posterior basisternal process***; along its anterior margins, the shield has a weakly delimited ***propleural contact surface***, which is overlapped by the propleurae in situ and ends laterally before the anterolateral corners of the basisternal inflection; situated posteromediad on the shield is the ***prodiscrimenal pit***, which is elliptical in outline, slightly longer than wide, deep, and margined by the transverse basisternal ridge; the shield's posterior margin is broadly sinuate. In posteroventral (Figure 5D) or posterior view (Figure 5F), the ***basisternal inflection*** bears a median longitudinal ***external basisternal crest***, which decreases in height posterad and medially separates the ***paramedian grooves of the basisternum***, which themselves more‐or‐less hook onto the medial contact surfaces of the basicoxae (Figure 5D). The ***profurcal arms*** are invaginations that are visible internally as a tubular convexity of the profurca; they have short ***basal stalks of the profurca***; dorsally, each arm divides into the ***anterior profurcal branch***, which ends anteriorly at the *propleural articulation*, and the ***posterior profurcal branch***, which extends dorsomedially, nearly meeting the branch of the other side. The ***dorsal profurcal lamella*** is developed between the anterior and posterior profurcal branches and expands dorsally; the left and right lamellae fuse dorsomedially, forming the dorsal portion of the ***profurcal bridge***, which forms the dorsal portion of the ***neural foramen***; each lamella is weakly concave across its surface, forming a shallow hyperbolic paraboloid (“pringle shape”), which curves most strongly posteromediad along the dorsal margin. The ***anterior profurcal apodemes*** are fused medially, forming the *anterior profurcal lamella*. The ***posterior profurcal lamellae*** of each side are directed dorsomedially, where they meet forming an acute angle and continue as a single lamella to the dorsal margin of the neural bridge. Posterad the profurcal branches, the furcasternal region is extended posteriorly as a distinct and comparatively long ***furcasternal lobe***; this lobe is marked at its anterior apices by the ***insertion scars of Ivlm7a***.

### 3.4 Oligopropodosoma

The mesothorax, metathorax, and propodeum are completely fused, forming the ***oligopropodosoma***.

**Figure 6:**
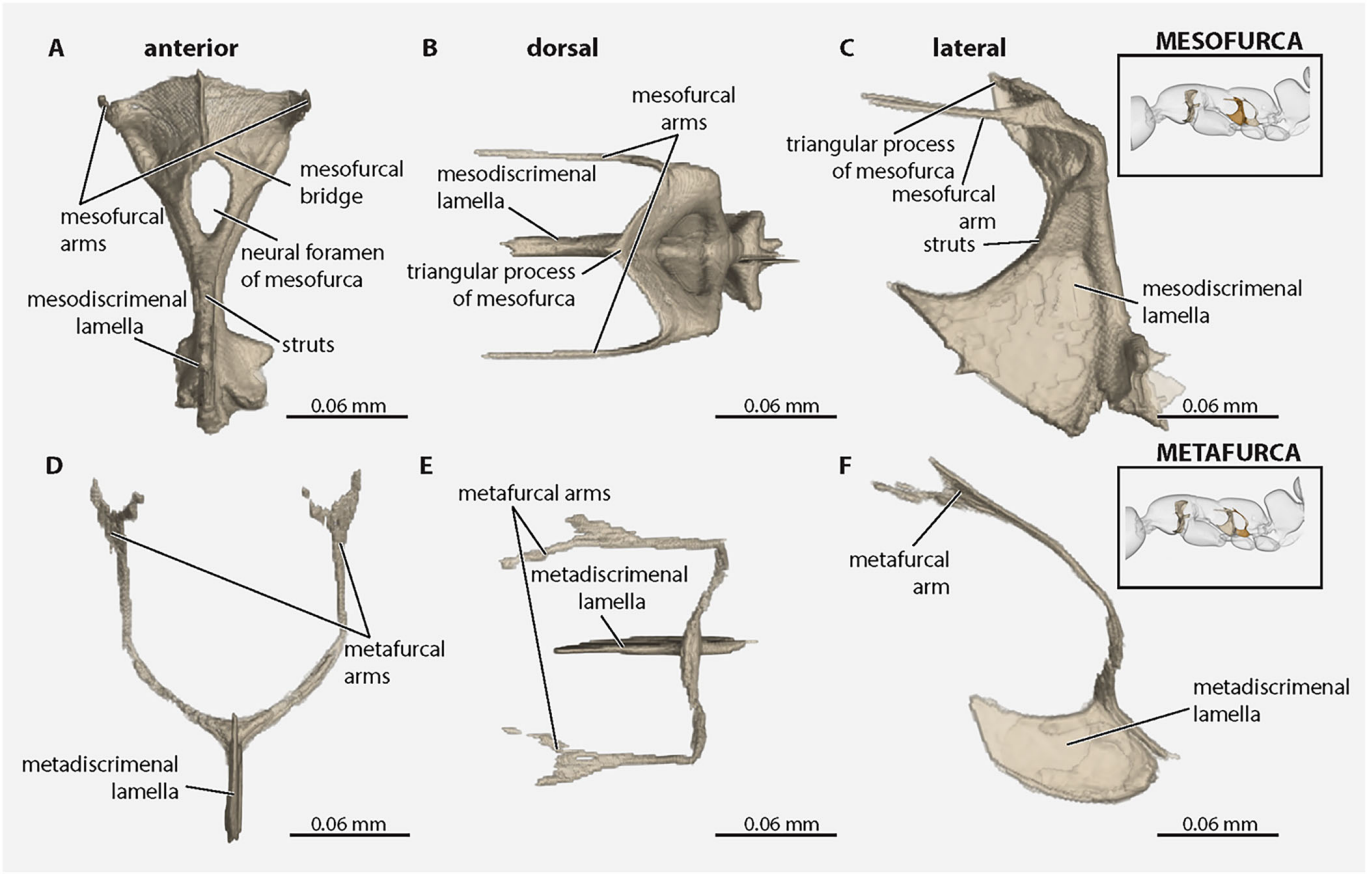
*Protanilla lini*, endoskeletal elements of the meso‐ and metathorax. (A–C) Mesodiscrimenal lamella and mesofurca; (A) anterior view; (B) dorsal view; (C) lateral view. (D–F) Metadiscrimenal lamella and metafurca; (D) anterior view; (E) dorsal view; (F) lateral view.

The **mesothorax** comprises the *mesonotum*, the *mesopectus*, and the *mesendosternum* (Figures 1 and 2A,C,D), which are completely fused, being distinguished anteriorly by the mesospiracular notches*, which are roughly circular in sagittal view (Figure 2B), and laterally by the ***mesonotopleural suture*** (=*notopleural suture*, HAO; nps2, Figure 2B), which is indicated by a series of short carinae; it is distinguished externally from the metathorax by the ***mesometapleural suture*** (nps2, Figure 2A), which itself is marked by a distinct carina that extends from near the *ventrolateral mesometathorarcic margin* anterodorsally to the rounded *posterolateral corner of the mesonotum*. Externally, the mesothorax is divided into anterior (ventral) and posterior (lateral) portions by the ***epicnemial carina***, which forms a complete U‐shaped margination, as it extends from the rounded anterolateral corner of the *mesopleural area of the mesopectus* along the anterolateral boundary of this area with the *sternal area of the mesopectus* up to and across the surface of the mesonotum; dorsad the *transverse mesopleural sulcus* and the rounded process of the epicnemial carina*, this carina is recognized as the dorsal epicnemial carina* (Figure 2A). The anterior portion of the mesothorax is the ***mesothoracic connexus*** *, which comprises the elongated mesonotal connectite* and the elongated ***mesopectal connectite*** * (Figure 2); internally, the connexus is distinguished from the non‐articulatory portion of the mesothorax by distinct surface curvature and the evagination of the epicnemial process* (eep, Figure 2B). Externally, the ***mesonotum*** (Figure 2A,C) is divided into the anterior *pronotal contact surface* (=external surface of the mesonotal connectite) and the posterior *mesonotal disc* by the epicnemial carina; laterally, its anterior portion is continuous with the *anterior mesopectal groove* and its posterior region with the *upper mesopleural area*; the disc is offset from the surrounding cuticle by wrinkled surface sculpturing. The ***mesopectus*** is divided into the anterior *mesopectal connectite* and the *ventral mesopectal area* by the *anterolateral mesopectal ridge*, and the ventral area is separated from the *mesopleural areas* by the epicnemial carinae. The ***mesopectal connectite*** is further divided into anterior and posterior portions by the anterolateral mesopectal ridges*, which are continuous with the anteromedian mesopectal ridge*, which itself divides the *anterior mesopectal flange* and *groove* into symmetrical halves, with both the anteromedian and anterolateral ridges forming the anterior margin of the anterior mesodiscrimenal pit* (Figure 2D). The mesopectal connectite* (=anterior portion of the *ventral mesopectal area*) is distinctly elongated, with each half of the anterior mesopectal groove being slightly shorter than wide. The ***anterior mesopectal flange*** (amf, Figure 2D) is distinctly produced anteriorly, concealing the apex of the furcasternal flange in ventral view with the procoxae removed; its surface is irregularly striate. The ***anterior mesopectal groove*** (Figure 2D) is paired, with almost regular transverse ridges. The ***posterior portion of the ventral mesopectal area*** has some nearly regular striations posterad the anterior mesodiscrimenal pit; it is otherwise smooth and is extended posteroventrally as the ***mesosternal processes***, which are low and rounded; posterad these weakly differentiated processes are the ***mesocoxal concavities***, which are laterad the ***mesofurcal pit*** and which surround the ***mesocoxal foramina***, with their distinct, raised periarticular mesocoxal rims* (pmsr, Figure 2D); the foramina bear the ***medial mesocoxal condyles*** slightly anterad their midlengths. The ***mesopleural area*** is divided into the depressed ***dorsal mesopleural area*** (dma) and the raised ***ventral mesopleural area*** (vma) (Figure 2A) by the ***transverse mesopectal sulcus*** (tms). The mesopleural area is separated from the ventral mesopectal area by the ***ventrolateral mesopectal carina*** (vlmspc, Figure 2A,D). Internally, the mesopectus bears the ***mesendosternum***, which comprises the anteromedially situated *mesodiscrimenal lamella* and posteromedially situated *mesofurca*, which themselves are physically continuous (Figures 3A, 4, and 5). The ***mesodiscrimenal lamella*** is dorsoventrally broad; it extends anterodorsally along the midline of the posterior mesopectal portion but is not indicated externally and ends posteroventrad the *mesodiscrimenal pit*; it is margined dorsally by the ***mesofurcal struts***, which form broad anterior braces of the *mesofurcal arms*. The ***mesofurca*** (Figures 3A, 4, and 6A–C) is divided into the ***mesofurcal arms***, each of which subdivides into the *lateral mesofurcal branch* and *medial mesofurcal branch*. Each ***lateral mesofurcal branch*** extends anteriorly as a long, narrow, threadlike processes. Each ***medial mesofurcal branch*** fuses dorsomedially, forming the ventral portion of the ***mesofurcal bridge***, above the *neural foramen of the mesofurca*. Between the lateral and medial branches of each furcal arm is the ***dorsal mesofurcal lamella***, which are directed anterolaterally and fused medially, forming the dorsal portion of the ***mesofurcal bridge***, above the *neural foramen of the mesofurca*. At the posterolateral bases of the mesofurca is the ***paracoxal ridge*** (=*transverse metapectal ridge*, Figure 3A), which is lateromedially short in anterior or posterior view and is thick in dorsal view.

The **metathorax** (Figures 1 and 2) comprises the undifferentiated *metanotal region*, the *metapectal region*, and the *metendosternum*. The ***metanotal region*** (Figures 1 and 2A,C) is represented externally by the transverse ***metanotal groove*** (i.e., the dorsal groove that corresponds to the position where the metanotum were to develop if it were to do so, see, e.g., Figure 3 of Boudinot, Khouri et al. 2022), which is delimited laterally by the closed ***metathoracic spiracles*** and is traversed by short, parallel, and longitudinally oriented carinulae. The ***metapectal region*** is divided into the *metapleural region* and the *metasternal region* by the ***ventrolateral metapectal carina*** (Figure 2A). The ***metapleural region*** is indistinguishable dorsally from the propodeum; these regions are distinguishable posterolaterally, by the ***metapleural gland bulla***, which forms a longitudinally oriented bulge ventrad the *propodeal spiracle* (Figure 2A). The metapleural gland evaporatorium* (mpge) is a longitudinal groove that is margined ventrally by the ventrolateral metapectal carina and dorsally by the ***dorsal flange of the metapleural gland evaporatorium*** (dfmpge); it extends anteriorly to the mesometapleural suture; it is broad posteriorly, being continuous with the metacoxal bridge, with this surface divided by the ***vertical subtending carina of the metapleural gland*** * (mpgvsc, Figure 2A,D) and is roughened by small, raised bumps and a few transverse carinulae. The ***metapleural gland orifice*** (mpgo, Figure 2D) is ovate and completely concealed by the dorsal evaporatorial flange. The ***sternal metapectal area*** is narrower than the respective area of the mesopectus. Medially on the metasternal area is the ***metafurcal pit*** (mtfp, Figure 2D), which is posterad the lateromedially undifferentiated and low and rounded ***metasternal bulge*** (mtstb, Figure 2D); it is surrounded laterally by weak impressions that form the ***metacoxal concavities*** (Figure 2D), and it borders the distinct and deep ***posteromedian metasternal depression***. The ***metacoxal foramina*** are margined similarly to those of the mesocoxae by the periarticular metacoxal rims* (pmtr, Figure 2A,D); they bear the ***medial metacoxal condyles*** anterad their midlength and are enclosed posteriorly by the *annulus* (*sensu* Bolton 2003) or ***metacoxal bridges***. The ***metendosternum*** comprises the *metadiscrimenal lamella* and the *metafurca* (Figures 3A and 6D–F). The short ***metadiscrimenal lamella*** is longer than it is tall; it is and curved along its dorsal and ventral margins; it meets the base of the mesofurca and lacks distinctly developed *metafurcal struts*. The ***metafurca*** is divided at its base into the very thin and long ***metafurcal arms***, which themselves are directed dorsolaterally before curving, where they become parallel and anteriorly directed before dividing apically.

The dorsal surface of the **propodeum** is offset from the mesonotum by the metanotal groove and is continuous with the metapleural area laterally; in profile view, it is longer than high, with the dorsal margin curving through a distinct yet broad convexity to the posterior margin (Figure 2A); it bears the circular ***propodeal spiracles*** near the anterodorsal margin of the metapleural gland bulla (Figure 2A). The posterior surface of the propodeum is flattened offset from the lateral surfaces by a shallowly convex margin (Figure 2A). The ***propodeal foramen*** is completely encircled by a flange, which is distinctly divided into the *lateral propodeal lobes* and the *dorsal propodeal lobe* as seen in posterior view (Figure 4A); anterad the foramen are a few transverse carinulae. The ***lateral propodeal lobes*** (lppdl, Figure 2A–D) are truncate in lateral view and form the posterior margin of the metapleural gland evaporatorium. The ***dorsal propodeal lobe*** (dppdl, Figure 2A–D) bears the paired and ventromedially directed ***propodeal condyles*** (ppdc, Figure 2B,D).

### 3.5 Legs

**Figure 7:**
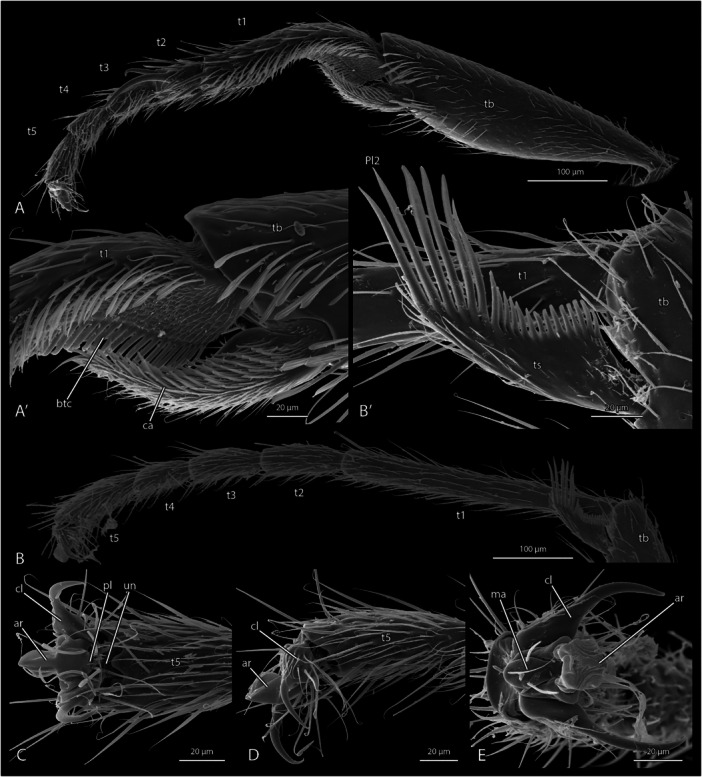
Scanning electron micrographs of the legs of *Protanilla lini*. (A) Tibia, tarsus and pretarsus of foreleg; (A’) antenna cleaner (strigil) of foreleg with calcar; (B) hind leg, apex of tibia with spur and tarsus; (B’) metatibial spur; (C–E) pretarsus. (A) anterior view; (B) dorsal view; (C) lateral view. **ar **= arolium, **btc **= basitarsal comb, **ca **= calcar, **cl **= pretarsal claw, **ma **= manubrium, **pl **= planta, **t1 **= basitarsus, **t2–5 **= tarsomeres 2–5, **tb **= protibia, **ts **= tibial spur, **un **= unguitractor.

The slender legs (Figures 1 and 7) are similar in their general configurations but differ in the length and the width of specific parts, their armature, and the details of their articulations. All three pairs bear a regular vestiture of fine setae of c. 50 µm length on most of their surface. The forelegs are equipped with a well‐developed strigil, which is a functional complex comprising the calcar and probasitarsus. The procoxae are about twice the size of their pterothoracic counterparts (Figures 1 and 3). The profemur is wider than the metafemur, which is again wider than the mesofemur. The tibia of the foreleg (Figure 7A) is the widest, but the hind tibia is longer. All tarsi are pentamerous, with the basitarsomere about as long as the four other tarsomeres combined (Figure 7A,B). The pretarsal claws and the arolia are well‐developed (Figure 7C–E). Tarsal plantulae are lacking on all legs.

The **procoxa** is about 0.4 mm long and has an elongate, rounded conical shape (Figures 1 and 3). The cuticle is smooth, without any microreticulation or other surface patterns; most of the surface bears the regular vestiture of fine setae, but the posterior side is glabrous. The glabrous articulatory area of the trochanter is slightly convex; the articulatory socket for the trochanter is enclosed by a low rim.

The short **protrochanter** (c. 0.08 mm) appears cup‐shaped (Figure 1), with a nearly straight anterior edge and a rounded posterior margin; the apical edge is also nearly straight. The surface is smooth and almost completely glabrous; a single short seta is inserted on the lateral surface. The articulatory basal piece of the trochanter is completely sunk into the coxal articulatory socket, and thus not visible.

The **profemur** (c. 0.48 mm long) (Figure 1) is distinctly larger than its pterothoracic counterparts; it is slightly narrowed basally, distinctly widened in the middle region (c. 0.17 mm), thus almost appearing club‐shaped, and then distinctly narrowed towards the apex; the dorsal side is evenly rounded, whereas a distinct edge is present along the ventral margin, fitting with the tibia in its resting position.

The **protibia** is about 0.4 mm long (Figure 7A); it appears club‐shaped, distinctly narrowed proximally, then distinctly widening, and only very slightly narrowed apically; it is slightly wider than the metatibiae but shorter; a deep emargination for the insertion of the ***calcar*** is present apicoventrally (Figure 7A′). The setation is regular on the tibial surface like on the femur; groups of specialized spatulate setae (c. 50 µm long) with longitudinal ridges are present above and proximad the apical emargination, oriented toward the well‐developed calcar. A regular vestiture of fine and short setae is present on the surface of the calcar, which is ca. 100 µm long, widening in its middle region, narrowing distally, and acuminate apically; a dense vestiture of short, spatulate microtrichia (c. 20 µm) is present on its inner surface; its articulatory membrane displays a field of extremely short microspines. A second apical tibial spur is not present.

The ***probasitarsomere*** of the five‐segmented **tarsus** (Figure 7A) is ca. 0.22 mm long, parallel‐sided, strongly curved basally, and also distinctly curved in its middle region; the basal articulatory piece connecting it with the articulatory socket of the apical protibia is separated by two transverse furrows from the main part of the segment; ventrally it bears a field of microspines, similar to those of the articulatory membrane of the calcar. The regular vestiture of fine setae of the probasitarsomere is largely restricted to the dorsal proximal region, but denser than on the other parts of the leg; a small field of minute scale‐like surface structures is present close to the articulation with the tibia; more widely spaced, longer and thicker setae are inserted on the anterior side of the distal half of the basiprotarsomere. Densely arranged spatulate tenant setae are inserted along the anteroventral edge; a row of regular setae is present above them, extending over about half the length of the tarsomere; the posteroventral edge bears a very dense row of parallel‐sided, flattened and apically truncated microtrichia (c. 30 µm long); a very dense vestiture is present on the surface between the anteroventral and posteroventral row, basally very short hair‐like microtrichia (ca. 5 µm) and more distad scale‐like structures with minute distal spines, both oriented towards the flattened microtrichia. Several strong setae (c. 40 µm) with a pattern of longitudinal riffles are inserted apicoventrally, and some finer and some medium sized setae subapically.

***Protarsomere 2*** is about 50 µm long, straight, and almost as wide as long. The regular vestiture of fine setae is present. A long (ca. 0.13 mm), strong and sinuate seta with longitudinal riffles is inserted apicolaterally, and a similar curved seta of about half the length above it. ***Protarsomere 3*** is slightly longer than 2 but less wide, widening slightly towards its apex. It bears the fine setation and few stronger setae distally. ***Protarsomere 4*** is similar. The apical ***protarsomere 5*** is ca. 60 µm long and moderately widened towards its apex; it bears the regular vestiture of fine setae.

The pretarsal claws are divided into a wide basal part, and an abruptly narrowing distal portion, both about equally long; the surface is smooth; several fine setae (ca. 8 µm) are inserted on the external side of the proximal part; a tooth is not present; an ***unguitractor plate*** is not recognizable in the scan data.

The well‐developed membranous *arolium* (Figure 7C–E) is almost as long as the claws; it is folded laterally, suggesting the presence of an internal ***arculus***; its apical edge has a finely crenulated surface structure; a distinct ***manubrium*** with a pair of fairly long setae (ca. 25 µm) is present above its dorsal half; a ***planta*** is not differentiated in surface structure relative to the arolium, but likely represented by an oval area blow it with a pair of proximolateral setae (Figure 7C).

The **mesocoxa** (Figure 3A) is about half as long as the procoxa and more globular but otherwise similar. A very short transverse accessory segment is present between the **mesotrochanter** and the **mesofemur** (Figure 1). The latter is shorter (ca. 0.32 mm) and less wide (ca. 80 µm) than the profemur. The **mesotibia** is as along as the mesofemur and moderately widened in its distal 3rd; the apex is obliquely truncated; tibial spurs are missing. The ***mesobasitarsomere*** is as long as the ***probasitarsomere*** but completely straight, and entirely covered with the regular vestiture of fine setae. The remaining tarsomeres are similar to their prothoracic counterparts but lack specialized setae. The claws and arolium are similar to the corresponding structures of the foreleg.

The **metacoxae** (Figure 3A) are about 0.24 mm long. The **metafemur** is longer and wider than the mesofemur. The **metatibia** (Figure 7B) is longer than the protibia but similar in its shape; apically it bears a complex flattened spur, c. 90 µm long, with a very dense mesal row of flattened microtrichia, apically truncated and ca. 12 µm on the basal half of the segment, and about three times longer more distally. The **metatarsus** (Figure 7B) is similar to the mesotarsus in its proportions and vestiture. The ***arolium*** is about as large as the arolia of the forelegs and middle legs but is parallel‐sided and has a simple cushion‐like structure.

### 3.6 List of Mesosomal Muscles of Ant Workers

The mesosomal muscle list is based on Lubbock (1881), Janet (1898), Snodgrass (1942), Saini et al. (1982), Liu et al. (2019), Markl (1966), and especially Aibekova et al. (2022). The nomenclature follows Friedrich and Beutel (2008) and Beutel et al. (2014), where Roman numerals refer to the segment (e.g., I = prothorax). The numbers used by Snodgrass (1942) for the muscles of the honeybee are given in brackets, or if the muscle is lacking in the honeybee the number used by Markl (1966) for *Formica*.

#### 3.6.1 Prothoracic Muscles

(Figures 8, 9, 10).

**Figure 8:**
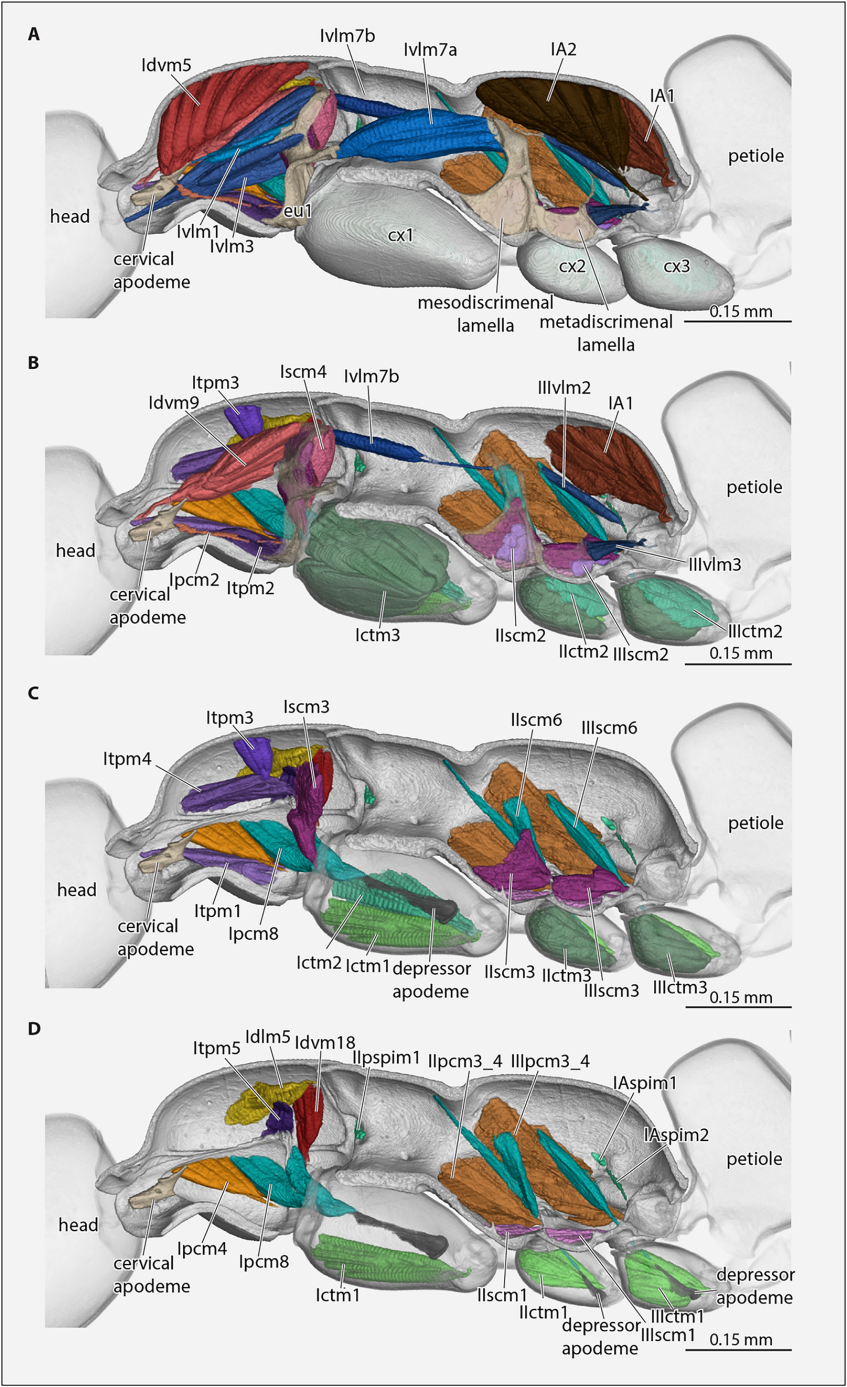
*Protanilla lini*, posterior head region, mesosoma with muscles, petiole (major part), all sagittal view. (A) With complete set of muscles; (B–D) layers of muscles successively removed, from mesal to lateral. **cx1–3 **= pro‐, meso‐, metacoxa. For muscle abbreviations, see the text.

**Figure 9:**
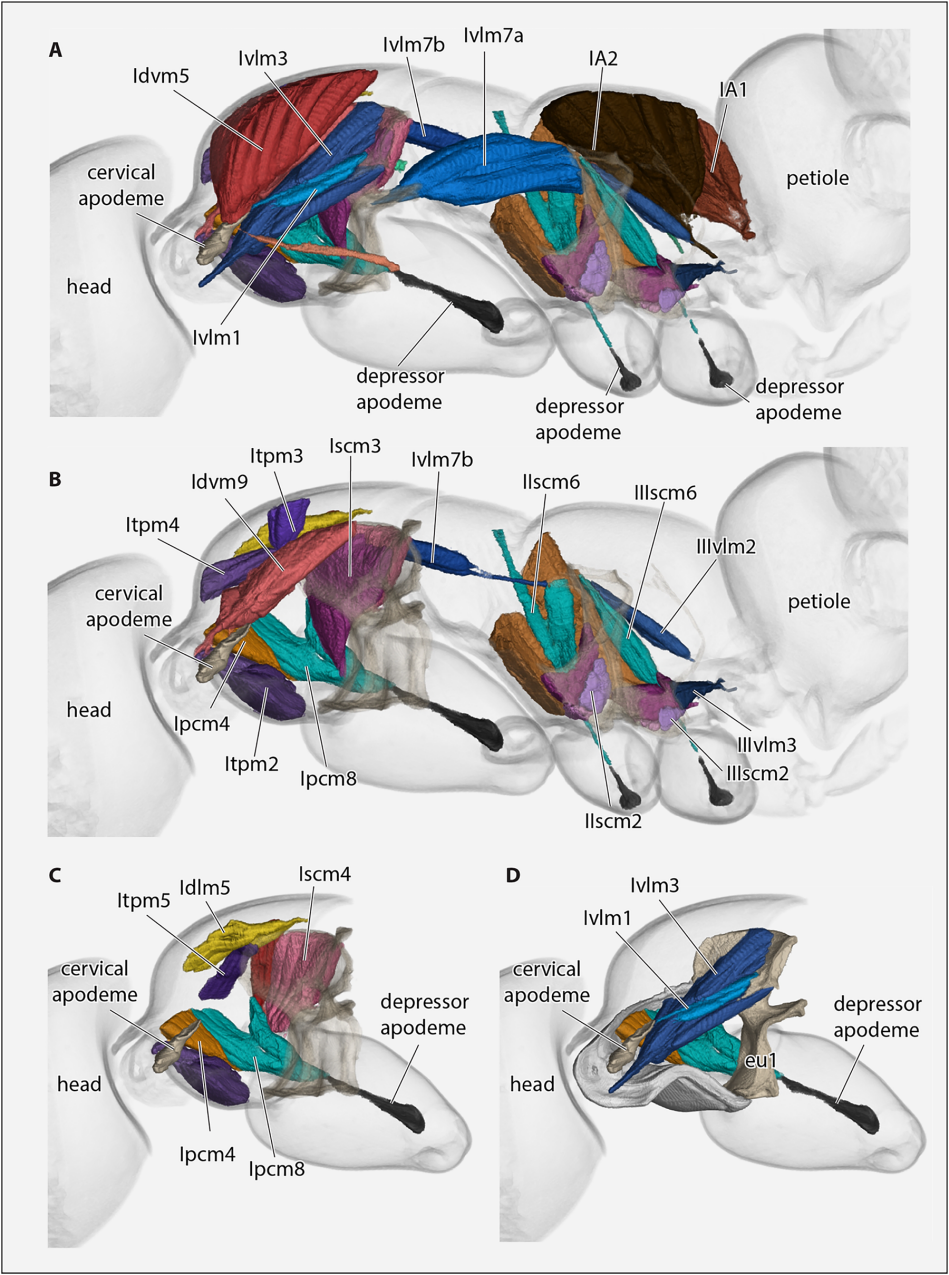
*Protanilla lini*, (A and B) posterior head region, mesosoma with muscles, petiole (major part), in sagittal view. (C and D) Posterior head region and prothorax with muscles. For muscle abbreviations, see the text. **cx1– 3 **= pro‐, meso‐, metacoxa, **eu1 **= prothoracic eusternum.

**Figure 10:**
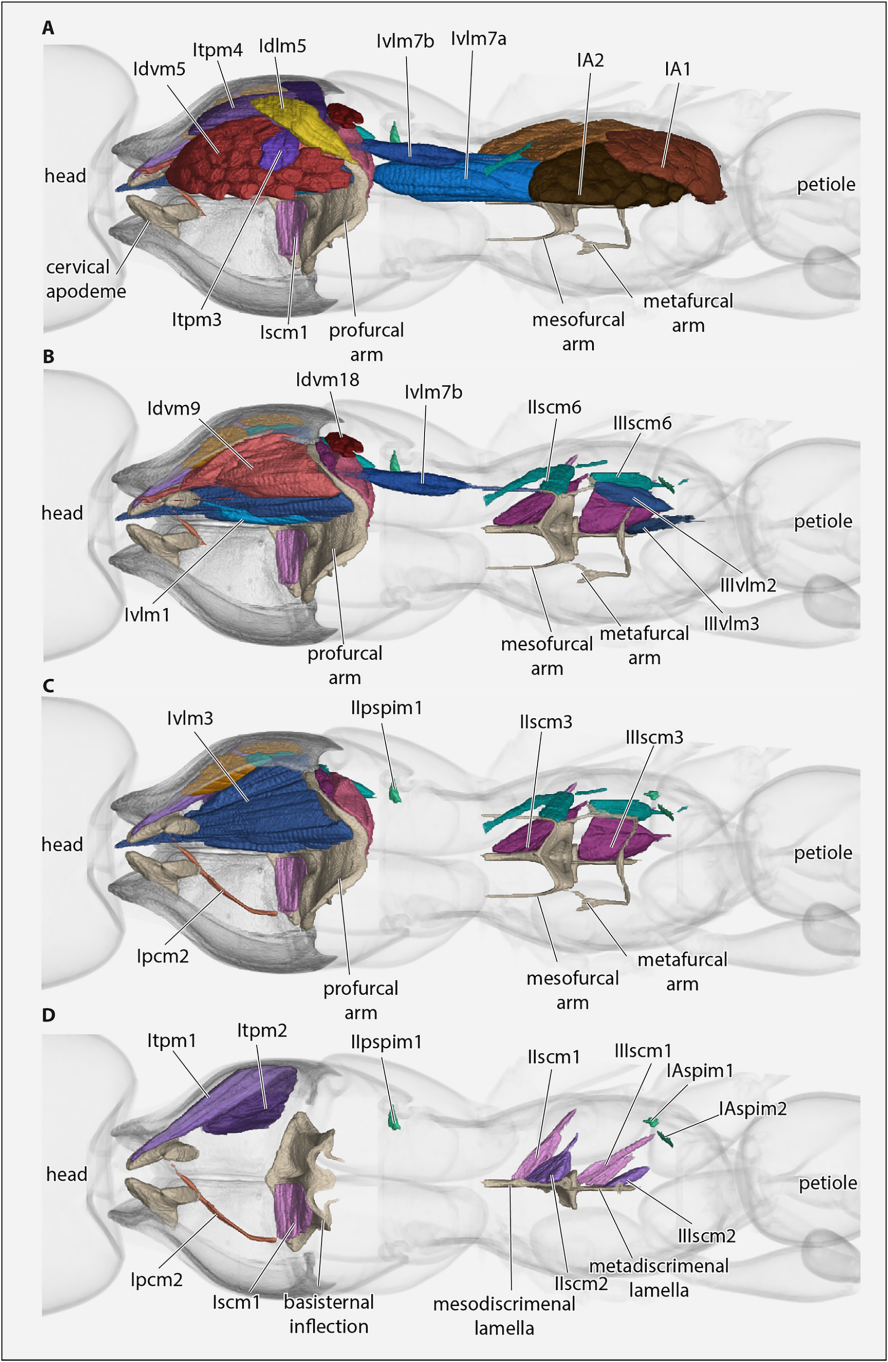
*Protanilla lini*, (A–D) posterior head region, mesosoma with endoskeletal structures and muscles, petiole (major part), all in dorsal view with cutice rendered semitransparent. For muscle abbreviations, see the text. **cx1–3 **= pro‐, meso‐, metacoxa; **eu1 **= eusternum.

##### 3.6.1.1 Ronoto‐Cephalic Muscles

(Figures 8A,B, 9A,B, and 10A,B).

*Levators of the head*.

**Idlm1**, *M. prophragma‐occipitalis* (40 + 41); a longitudinal dorsal cervical muscle. Absent in *Protanilla* (Figures 8, 9, 10).

**Idvm9**, *M. profurca‐occipitalis* (43); a large cervical muscle with a broad origin and converging on a narrow tendon (Figures 8B and 9B). **O**: on the anterior face of the dorsal portion of the profurcal arm; **I**: dorsolaterally on the postocciput. *Functional note*: levator of the head if symmetrically contracted or rotator if asymmetrically contracted.

**Propleuro‐cephalic muscles**

*Levators of the head*

**Itpm1**, *M. pleurocrista‐occipitalis* (42a); a cervical muscle with a moderately broad area of origin and converging towards its insertion (Figure 8C). **O**: laterally on the propleuron; **I**: dorsolaterally on the postocciput with a tendon. *Functional note*: Levator or rotator, like Idvm9.

**Itpm2**, *M. propleuro‐occipitalis* (42b&c); a medium sized, fan‐shaped extrinsic head muscle with a fairly broad area of origin (Figure 9D). **O**: ventrally on the propleuron below Itpm1, close to the posterior propleural margin; **I**: dorsolaterally on the postocciput, with its tendon attached very close to that of Itpm1. *Functional note*: levator or rotator like Idvm9.

**Profurco‐cephalic muscles** (Figure 9A)

*Depressor of the head*.

**Ivlm3**, *M. profurca‐tentorialis* (44); a large extrinsic head muscle (Figure 8A). **O**: surface of the dorsal propleural ridge and anterior surface of profurcal arms. **I**: ventromesally on the margin the foramen occipitale with a strongly developed tendon. *Functional note*: contraction pulls the head downwards.

##### 3.6.1.2 Pronoto‐Propleural Muscles

(Figure 8C,D)

*Pronoto‐propectal stabilizers*

**Itpm3**, *M. pronoto‐pleuralis anterior* (48); a moderately sized conical, nearly vertical muscle (Figure 8B,C). **O**: dorsally on the middle region of the pronotum, laterad Idvm5; **I**: mesally directed projection of the posterodorsal propleural region (see Itpm4). *Functional note*: interpretation from Markl (1966) (see also Aibekova et al. 2022).

**Itpm4**, *M. pronoto‐apodemalis anterior* (49); a moderately sized nearly horizontal muscle (Figure 8B). **O**: anterolaterally on the pronotum; **I**: apex of the profurcal arm and the propleural apophysis (pleural arm). *Functional note*: interpretation from Markl (1966); according to Snodgrass (1942) a protractor of the propectus.

*Propleural levator/rotator*

**Idvm5**, *M. pronoto‐occipitalis* (Markl 1966: 46 + 47); a large conical to fan‐shaped muscle (Figure 8A). **O**: on a large area of the internal surface of the pronotum, almost reaching the posterior margin; **I**: on the apical region of the cervical apodeme. *Functional note*: pulls the cervical apodeme and anterior propleuron towards the pronotum (Duncan 1939). *Homology note*: This muscle moves the head via a free cervical sclerite in most other groups of insects (e.g. Beutel et al. 2014).

##### 3.6.1.3 Pronoto‐Profurcal Muscle (Figure 8D)

*Profurcal stabilizer*

**Itpm5**, *M. pronoto‐apodemalis posterior* (Markl 1966: 50); band‐shaped, slightly oblique, nearly vertical (Figure 9C). **O**: lateral margin of the pronotum; **I**: lateral margin of the posterior profurcal surface.

**Propleuro‐profurcal muscle** (Figures 8A, 9A, and 10B)

*Profurcal stabilizer*

**Ivlm1**, *M. profurca‐cervicalis* (51); a slender cervical muscle converging towards its apex (Figure 8A). **O**: mesally on the anterior surface of the profurcal arm and anterior profurcal lamella; **I**: a small area on the posterior apex of the cervical apodeme.

**Procoxal muscles** (Figures 8B–D and 9B,C)

*Remotors of the procoxa*

**Idvm18**, *M. pronoto‐coxalis lateralis* (55); a moderately sized, rather slender and conical muscle of the procoxa (Figure 10B). **O**: dorsolaterally on the posterior pronotal region; **I**: posterolaterally on the lateral procoxal process.

**Iscm4**, *M. profurca‐coxalis lateralis* (56); a large extrinsic coxal muscle (Figure 8B). **O**: posteriorly on the profurcal arm and posterior profurcal lamella; **I**: posterolaterally on the lateral procoxal process, slightly below the insertion area of of Idvm18.

*Rotators of the procoxa*

**Ipcm2**, *M. procoxa cervicalis transversalis* (Markl 1966: mcr, M. cruciatus); a pair of thin and curved extrinsic procoxal muscles that cross over one another medially (Figures 8B and 10C,D). **O**: posterior apex of the cervical apodeme; **I**: anterolaterally on the lateral procoxal process on the other side of the body. *Functional note*: in addition to remoting the procoxa, also stabilizes the prothorax according to Markl (1966), even though the effect is likely minimal considering the very small diameter of the muscle.

**Iscm1**, *M. profurca‐coxalis anterior* (Markl 1966: 54/2); a compact, short and parallel‐sided extrinsic coxal muscle (Figure 10A,D). **O**: laterally on the basisternal prodiscrimenal lamella; **I**: anterolaterally on the lateral procoxal process. *Functional note*: rotates the procoxa anterolaterad (Markl 1966).

*Promotors of the procoxa*

**Iscm3**, *M. profurca‐coxalis medialis* (Markl 1966: 54/1); a large, bipartite extrinsic coxal muscle (Figures 8C and 9B). **O**: posterior surface of the profurcal arm and profurcal stem; **I**: mesally on the procoxal rim.

**Ipcm4**, *M. propleuro‐coxalis superior* (53); a medium‐sized triangular extrinsic procoxal muscle (Figures 8B and 9C). **O**: ventral surface of the anterior propleural ridge; **I**: anterolaterally on the lateral procoxal process.

##### 3.6.1.4 Protrochanteral muscles

*Trochanteral depressors*

**Ipcm8**, *M. propleuro‐trochanteralis* and/or Iscm6, *M. profurca‐trochanteralis* (61); a well‐developed bipartite extrinsic leg muscle (Figure 9B,C). **O**: posteriorly on the ventral surface of the propleura and laterally on the basal portion of the profurca; **I**: protrochanteral depressor apodeme. *Functional note*: according to Markl (1966), this muscle is an important remotor of the proleg and also a depressor of the trochanter. *Homology note*: due to the areas of origin on two skeletal elements this muscle could be homologous with *M. propleuro‐trochanteralis* or *M. profurca‐trochanteralis*, or possibly with both.

**Ictm3**, *M. procoxa‐trochanteralis medialis* (62); the largest intrinsic procoxal muscle, bipartite and placed medially (Figure 8B; Model 1, 2, 6). **O**: anterior, posterior, and mesial procoxal surfaces, covering up to ¾ of the inner surface area; **I**: depressor apodeme of the trochanter, together with Ipcm8 (61).

*Trochanteral levators*

**Ictm1**, *M. procoxa‐trochanteralis anterior* (59); about half as voluminous as Ictm3 (Figure 8C,D). **O**: anterodorsal procoxal surface; **I**: base of the trochanter.

**Ictm2**, *M. procoxa‐trochanteralis posterior* (60); triangular, about as large as Ictm1 (Figure 8C). **O**: dorsolaterally on the procoxal wall; **I**: base of the trochanter, close to Ictm1.

##### 3.6.1.5 **Intersegmental muscles** (Figure 9C)

*Prothoracic depressors*

**Idlm5**, *M. pronoto‐phragmalis anterior* (45); a medium‐sized fan‐shaped intersegmental muscle (Figure 8D). **O**: dorsolateral surface of the pronotum at about midlength; **I**: anterior mesonotal margin. *Functional note*: depressor of the pronotum, also stabilizes the connection with the mesonotum (Snodgrass 1942; Markl 1966; see also Aibekova et al. 2022).

**Ivlm7a,** *M. mesofurca‐profurcalis* (52/1); a large intersegmental muscle, posteriorly rather cylindrical in shape but anteriorly converging towards a strongly developed tendon (Figure 10A). **O**: anteriorly on the mesofurcal arm; **I**: posterolaterally on the dorsal margin of the furcasternum with a tendon. *Functional note*: a depressor of the prothorax according to Lubbock (1881, Plate XI, fig.2: m), and possibly also a retractor of the profurca (Kéler 1955, 74. Musculus mesofurcaprofurcalis; see also Aibekova et al. 2022).

**Ivlm7b**, *M. profurca‐mesofurcalis* (52/2); distinctly less thick than Ivlm7a, parallel‐sided anteriorly and converging toward a long tendon posteriorly (Figure 10A,B). **O**: laterally on the posterior surface of profurcal arm; **I**: apicolaterally on the mesofurcal arm with a tendon. *Function*: stabilizes the connection between the pterothoracic segments, prothoracic retractor, and elevator of the prothorax according to Lubbock (1881, Plate XI, fig.2: n).

#### 3.6.2 Mesothoracic Muscles

(Figures 8, 9, 10).

##### 3.6.2.1 Mesocoxal Muscles

(Figure 8B,C).

*Promotors of the mesocoxa*

**IIpcm3/4**, *M. mesanepisternalis‐coxalis* (80); a large and fan‐shaped lateral mesocoxal muscle (Figure 8D). **O**: on the anteroventral region of the mesopectus; **I**: anterolaterally on the mesocoxal rim.

**IIscm1**, *M. mesofurca‐coxalis anterior* (Markl 1966: 81); one of the three furca‐coxal muscles (Figure 8D). **O**: ventrally on the mesodiscrimenal lamella; **I**: anterolaterally on the mesocoxal rim. *Functional note*: interpretation as promotor from Snodgrass (1942).

*Remotors of the mesocoxa*

**IIscm2**, *M. mesofurca‐coxalis posterior* (83); a rather small fan‐shaped muscle that strongly converges toward its tendon (Figures 8B and 9B). **O**: mesodiscrimenal lamella and mesoventral portion of the mesopectal region; **I**: posteromesally on the mesocoxal rim.

**IIscm3**, *M. mesofurca‐coxalis medialis* (83); a medium‐sized large muscle that originates above IIscm2 (Figure 8C). **O**: on the mesodiscrimenal lamella; **I**: mesially on the mesocoxal rim.

##### 3.6.2.2 Mesotrochanteral Muscles

*Depressors of the trochanter*

**IIscm6**, *M. mesofurca‐trochanteralis* and/or IIpcm5, *M. mesanepisterno‐trochanteralis* (86); a bipartite trochanteral muscle (Figures 8B, 9B, and 10B). **O**: laterally on the mesofurcal arm and with a long and thin bundle on the anterodorsal mesopleural region; **I**: trochanteral depressor apodeme.

**IIctm3**, *M. mesocoxa‐trochanteralis medialis* (87, 88); the largest of the three coxa‐trochanteral muscles (Figure 8C). **O**: mesal mesocoxal wall, covering as large proportion of the internal surface; **I**: on the depressor apodeme of the trochanter, together with IIscm6 and IIpcm5.

*Levators of the trochanter*

**IIctm1**, *M. mesocoxa‐trochanteralis anterior* (84); a moderately sized fan‐shaped anterior mesocoxal levator (Figure 8D). **O**: anterior mesocoxal wall; **I**: anteriorly on the trochanteral base.

**IIctm2**, *M. mesocoxa‐trochanteralis posterior* (85); a moderately sized fan‐shaped posterior mesocoxal levator (Figure 8B). **O**: posterior mesocoxal wall; **I**: posterior trochanteral process, close to IIctm1.

##### 3.6.2.3 Intersegmental Muscles

**IIvlm3**, *M. mesofurca‐metafurcalis* (Markl 1966: 79); not identified, apparently absent.

##### 3.6.2.4 Muscles of the Metathorax and Propodeum

**Metacoxal muscles** (Figure 8B,C)

*Promotors of the metacoxa*

**IIIpcm3/4**, *M. metanepisternalis‐coxalis* (103); a very large, lateral coxal muscle (Figure 8D). **O**: laterally and ventrally on the metapleural region, extending to the lateral wall of the propodeum; **I**: laterally on the metacoxal rim.

**IIIscm1**, *M. metafurca‐coxalis anterior* (104); a moderately sized, fan‐shaped and flat muscle (Figure 10C). **O**: anteroventrally on the metapleural region and on the base of the metadiscrimenal lamella; **I**: anterolaterally on the metacoxal rim.

*Remotors of the metacoxa*

**IIIscm2**, *M. metafurca‐coxalis posterior* (106); a small spindle‐shaped muscle (Figures 8B and 9B). **O**: metadiscrimenal lamella below IIIscm3; **I**: posteromesially on the metacoxal rim.

**IIIscm3**, *M. metafurca‐coxalis medialis* (106); a relatively large metacoxal muscle (Figures 8C and 10C). **O**: posteriorly and laterally on the metafurcal arm, metadiscrimenal lamella, and paracoxal ridge (i.e., the transverse metapectal lamella of metapleural region); **I**: mesally on the metacoxal rim.

##### 3.6.2.5 Metatrochanteral Muscles

*Depressors of the trochanter*

**IIIscm6**, *M. metafurca‐trochanteralis* and/or IIIpcm5, *M. metanepisterno‐trochanteralis* (109); a well‐developed, spindle‐shaped trochanteral depressor (Figures 8B, 9B, and 10B). **O**: laterally on the metafurcal arm; **I**: trochanteral apodeme.

**IIIctm3**, *M. metacoxa‐trochanteralis medialis* (110); a well‐developed compact muscle, converging towards its insertion; (Figure 8C). **O**: ventromesal metacoxal wall, covering up a large proportion of the surface; **I**: trochanteral depressor apodeme, together with IIIscm6.

**IIIctm1**, *M. metacoxa‐trochanteralis anterior* (107); fan‐shaped anterior trochanteral levator, slightly larger than its mesothoracic equivalent (Figure 8D). **O**: anterior metacoxal wall; **I**: anterior trochanteral process (Markl 1966: Trochanterzapfen).

**IIIctm2**, *M. metacoxa‐trochanteralis posterior* (108); a well‐developed conical muscle (Figure 8B). **O**: posterior metacoxal wall; **I**: posterior trochanteral process, insertion close to IIIctm1.

##### 3.6.2.6 Spiracular Muscles

(Figure 8D)

*Occlusors of the spiracles*

**IIpspim1**, *M. mesanepisterno‐spiracularis* (73); a very small, slender muscle (Figures 8D and 10D). **O**: anterior margin of upper mesopleural area; **I**: small plate close to orifice of mesothoracic spiracle.

**IIIpspim1**, *M. mesopleura‐spiracularis* (Markl 1966: Ɛ); not identified.

**IAspim1**, *M. spiracularis I superior* (122); a very small muscle (Figure 9D). **O**: sclerotized bar above the propodeal spiracle; **I**: sclerotized bar below the propodeal spiracle.

*Functional note*: dilator of the propodeal spiracle.

**IAspim2**, *M. spiracularis I posterior* (123); very thin and slightly longer than IAspim1 (Figure 8D). **O**: skeletal bridge posterior to propodeum, between coxal and petiolar orifices; **I**: sclerotized bar below the propodeal spiracle.

##### 3.6.2.7 Muscles of Propodeum

(Not covered in Friedrich and Beutel 2008.)

(Figures 8A,B, 9A, and 10A)

*Levators of the petiole*

**IIIvlm3,** *M. metafurca‐abdominosternalis inferior* (119); a small, conical, horizontally oriented muscle (Figure 8B). **O**: metadiscrimenal lamella; **I**: ventrolaterally on the anterior margin of the petiole. *Functional note*: levator of the petiole and gaster under symmetrical contraction or rotator under asymmetrical contraction.

**IA1**, *1st elevator of abdomen* (Snodgrass 1942: 121; Markl 1966: 120); this large and conical muscle is the mesal elevator of the petiole (Figure 8A,B). **O**: on a large dorsomedial area of the propodeum; **I**: levator process of the petiolar tergum via an angled tendon.

**IA2**, *2nd elevator of abdomen* (Snodgrass 1942: 120; Markl 1966: 121); this very large and conical muscle is the second elevator of the petiole (Figure 8A). **O**: on a large dorsal area of the propodeum, laterad IA1; **I**: laterally on the membrane connecting the petiole with the posterior propodeal orifice via a short tendon.

*Depressor of the petiole*

**IIIvlm2,** *M. metafurca‐abdominosternalis superior* (118); a slender oblique longitudinal muscle (Figure 8B). **O**: posteriorly on the upper portion of the metafurcal arm; **I**: mesoventrally on the anterior margin of the petiole.

## 4 Discussion

Reconstruction of the morphological groundplan of Formicidae has been a multigenerational challenge, most effectively marked by the hypotheses of Bolton (2003: Appendix II). The primary limitation for the past 200 years has been unclear phylogeny, which led to many conflicting views on the ancestry and major patterns of morphological diversification among the ant subfamilies (see Keller 2011). This limitation has been largely resolved due to consensus mediated by the overwhelming informational content of genomic data, although some phylogenetic uncertainties persist (see Borowiec et al. 2025; Oberski et al. in press). The second limitation has been technology, as the difficulty of precise and high‐resolution (i.e., micron‐scale) observation severely increases with the taxon sample due to the manual effort required for specimen preparation, imaging, and depiction. The solution to this bottleneck is the digitization of ant anatomy, through efforts such as the scanning electron microscopy (SEM) atlas of Keller (2011), the macrophotographic image‐stacking database and portal of AntWeb (antweb.org), and increasingly the application of microcomputed tomography (µ‐CT). Because these technological approaches produce large quantities of data, and as these data can be processed further for quantitative or qualitative analysis, the pursuit of morphological digitization may be encapsulated as phenomics (Deans et al. 2012; Maddison 2016; Sigwart et al. 2025). Here, we take the big‐data morphology or phenomic approach through conventional and high‐throughput synchrotron µ‐CT to assess the anatomy of a key lineage of Formicidae: Leptanillinae, as represented by *Protanilla lini*, a member of the *P. taylori* species group (Borowiec et al. 2011; Griebenow 2024). We first address basic plesiomorphies and several new anatomical concepts derived from critical observation of *P. lini* (Section 4.1), before discussing apomorphies of *P. lini* itself (Section 4.2), and apomorphies of the clade Leptanillomorpha (Section 4.3).

### 4.1 *Protanilla* and the groundplan of Formicidae

A series of features observed here in *Protanilla* likely belong to the formicid groundplan, including the shield‐like pronotum, ball‐and‐socket‐like prothoracic‐postoccipital articulation (Aibekova et al. 2022), a ventral prothoracic complex mainly composed of freely movable propleural halves and a complex profurca, distinct proepisternal and proepimeral regions, and elongated procoxae with a specialized coxotrochanteral articulation (Boudinot 2015; Aibekova et al. 2022). All skeletal elements of the flight apparatus are reduced as in all ant workers (e.g., the notal wing processes, axillary sclerites, basalare, and subalare), which is a derived groundplan feature of the Formicoidea, i.e., the Formicidae in the widest sense (e.g., Boudinot, Khouri et al. 2022). The mesosomal skeletomusculature does not show modifications unequivocally linked with small size or hypogaeic habits—tendencies for which the Leptanillomorpha are known. As in other Leptanillomorpha, the mesosoma of *Protanilla* appears very slender in dorsal view, which arguably facilitates efficient motion in narrow hypogaeic spaces (Rabeling et al. 2008: Figure 2). However, a similar condition also occurs in other ants (Keller 2011), including the presumably epigaeic stem‐group representative †*Gerontoformica* (Sphecomyrminae) (Boudinot, Richter et al. 2022), and relative mesosomal breadth is highly variable in Formicidae (e.g. Keller 2011: fig. 22).

The most important potential plesiomorphies of the Formicidae revealed by the current study of *Protanilla* are unequivocally the “ornamentation” and regionalization of the mesosomal sclerites. We observe that the pronotum is margined by a distinct carina (the marginal pronotal carina*), which delimits the medial pronotal disc* and the lateral pronotal hypomeron*. The pronotal hypomeron is an external, differentiated region of the pronotal flange*, which bears on its medial surface the pronotal inflection. The pronotal hypomeron also curves around the procoxal bases posteriorly, likely restraining the motion of the coxae during locomotion or other activity. The pronotal flange is also divided into *anterior*, *ventrolateral*, *posterolateral*, and posterodorsal regions* by the *anterolateral*, *posterolateral*, and posterodorsal corners of the pronotum*, which are best visible in internal view, but are also indicated externally by the anterolateral and posterolateral angles or processes of the pronotal margin, and the dorsal corner of the pronotal lobe. Unlike in other ants, the anterolateral corner of the pronotum is vestigial, being scarcely visible as a weak curve of the pronotal ledge; hence, the anterior and ventrolateral regions of the pronotal flange are more‐or‐less continuous, which is a derived condition of *Protanilla*. Similar patterns are also visible for the propleurae, although we reserve focus on these bilateral homologs for future study.

Groundplan or at least plesiomorphic conditions of the worker mesothorax are neatly shown in *Protanilla* due to the large size of this segment and its ornamentation. We observe that the mesothorax is divided into anterior and posterior regions by the *epicnemial carina*, which completely encircles the segment. Because the portion of the epicnemial carina dorsad the transverse mesopleural sulcus may be absent, we recognize this specifically as the dorsal epicnemial carina*. While the convex pronotal contact surface of the mesonotum and depressed anterior extension of the mesopectus are both visible in external view, they are much more easily distinguished in sagittal view. In this view, both of these regions are offset by distinct surface curvature plus the evagination of the epicnemial process*. The dorsal and ventral portions of this distinct region—the *mesonotal* and mesopectal connectites*—are also distinguished by the mesospiracular notch*. In external view, the connectites are visible as the differentiated, tubular‐appearing, anterior portion of the mesothorax, which we conceive of as the mesothoracic connexus* (the articulatory region of the mesothorax, in more relaxed terms). The connexus is visible in some form or another across Formicidae, whether or not it is well‐developed, reduced, or otherwise concealed.

**Figure 11:**
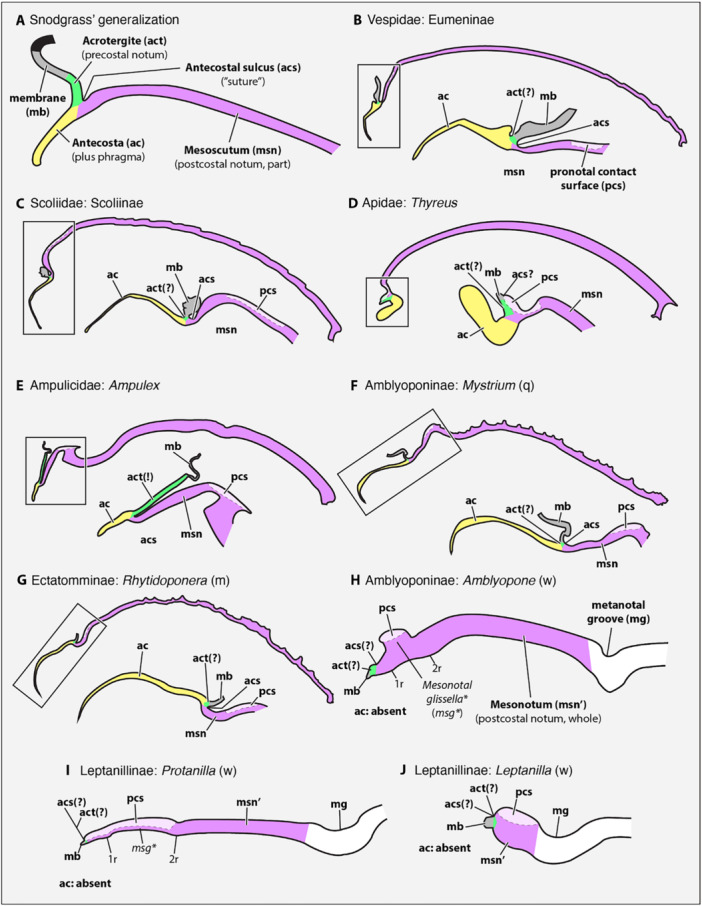
Homologies of the mesonotal connectite, represented by medial or slightly paramedial sagittal sections of the mesonotum. Not to scale. (A) The generalization of Snodgrass (1935, p. 162) for the “intersegmental sclerotization” of the mesonotum, where the acrotergite bears the intersegmental membrane anteriorly and the antecosta corresponds positionally to the transverse antecostal sulcus (“antecostal suture”). Representative non‐ant Aculeata (B–E), representative alate ( = winged) ants (F and G), and representative worker ants (H–J). *Thyreus* (D) redrawn from Meira et al. (2024) and *Amblyopone* (H) redrawn from Lieberman et al. (2022). **1r, 2r **= first and second ridges from Figure 2B; **m **= male; **q **= queen; **w **= worker;

The mesothoracic connexus—the anterior articulatory complex of the mesothorax—is newly recognized here, hence its interfamilial homologies need to be considered. For the mesonotal connectite, the question is whether the portion that bears the dorsal (external) contact surface (N2‐pcs in Figure 2A,C) corresponds to any portion of the “intersegmental sclerotization” of Snodgrass (1935, 161), and whether either of the internal and broadly rounded ridges correspond to the antecosta (1r, 2r in Figure 2B). Based on comparison to published (Apidae: *Thyreus*, Meira et al. 2024; Amblyoponinae: *Amblyopone*, Lieberman et al. 2022) and additional SR‐µ‐CT data (Table 1) across the Aculeata, we can resolve this homology problem (Figure 11). The “intersegmental sclerotization” of Snodgrass comprises the acrotergite (act, Figure 11A), the antecosta (ac, Figure 11A), and the “antecostal suture” (acs, Figure 11A). We observe that in Vespidae, Scoliidae, Apidae, and alate Formicidae (Amblyoponinae, Ectatomminae) the acrotergite is reduced and not developed as a distinct, sclerotized region of the mesonotum (Figure 11B–D,F,G). In *Ampulex*, however, the acrotergite is large and conspicuous (Figure 11E). In the sampled worker ants (Figure 11H–J), it appears that the entire “intersegmental sclerotization” complex is reduced, as there is no invaginated sclerotization ventrad the intersegmental membrane (hence the antecosta is absent), and the acrotergite is plesiomorphically reduced.

We further observe that the pronotal contact surface of worker ants forms a differentiated, transverse carina (Figures 2C and 11H,I). Internally, we observe that this carina is a transverse evagination that is margined by a pair of transverse, very low, rounded ridges (1r, 2r, Figures 2B and 11H,I). Although the posterior margin of this contact‐surface‐bearing region is continuous with the dorsal epicnemium* (depcnc, Figure 2B), it corresponds with an evagination, whereas the epicnemium is a carina, restricted to the cuticular surface. For this reason, we conceive of the elevated region of mesonotum in worker ants that bears the pronotal contact surface as the mesonotal glissella* (etymology: Proto‐Germanic: “glidan” or “to glide”) (msg, Figure 11H,I). It is apparent that the glissal region* can be short (*Amblyopone*, Figure 11H), elongate (*Protanilla*, Figure 11I), or undifferentiated from the rest of the reduced mesonotum (*Leptanilla*, Figure 11J). Promesonotal fusion is widespread among Formicidae (e.g., Bolton 1994, 2003; Keller 2011), hence we expect that in such cases the entire mesonotal connectite is undifferentiated.

Unlike the mesonotal connectite, the homologies of the mesopectal connectite remain a challenge outside of the scope of the present study. To resolve the homology problem of the mesopectal connectite would require solving the fate of the prepectal sclerites in Formicidae. Until a study makes dedicated comparisons of worker ants to males, queens, and non‐ant Hymenoptera—with particular reference to musculature (see, e.g., Meira et al. 2024, especially figure 22 therein)—we prefer to leave this problem to future work. In the meantime, we encourage the usage of the terms “mesothoracic connexus” and “mesopectal connectites” in a homology‐neutral manner if comparisons are made outside of Formicidae, and in a homology‐explicit manner within the ants, in analogy to Bolton's concept of metasomal presclerites (Bolton 1990; compare to the graduli of Apoidea, see, e.g., figure 33 of Meira et al. 2024). The new term “mesonotal connectite” may be used to refer in a homology‐explicit manner for the “intersegmental sclerotization” (*sensu* Snodgrass) of the mesonotum, recognizing the mesonotal glissella as necessary. We further observe that the connexus and connectite concepts will have utility when the diversity of promesothoracic fusion across Formicidae is reevaluated (see, e.g., Bolton 1994, 2003; Keller 2011; Richter et al. 2023).

Finally, although the metathorax of *Protanilla* appears unremarkable, the metapleural gland region deserves special attention. Our renders of *Protanilla*, particularly in comparison to *Amblyopone* (Figures 3 and 4 of Lieberman et al. 2022, supplemented by the SEM atlas of Keller 2011 and other data), clarify several key anatomical facts otherwise obscured by the cuticular simplification of *Formica* (see, e.g., Figure 2 of Aibekova et al. 2022). We observe that the mesopleural area of *Protanilla* above the ventrolateral metapectal carina (vlmtpc, Figures 2A,D and 4A,C) and below the dorsal metapleural gland flange (dfmpge, Figures 2A and 4A,C) is “trough‐like” as described by Borowiec (2016) for Dorylinae. This sulcus extends from the external orifice of the metapleural gland (mpgo, Figure 2D) and reasonably forms a surface across which would spread a seeping liquid from the ventrally directed gland orifice. For this reason, we have here termed this the metapleural gland evaporatorium* (mpge, Figures 2A and 4A,C). Across the ants, the evaporatorium is variable in length, sculpture, and margination. Below and posterad the metapleural gland orifice is a carina arising from the periarticular metacoxal rim* that extends posterodorsally onto the propodeal lobe (lppdl), and which we name the *vertical subtending metapleural gland carina* * (mpgvsc, Figures 2A and 4A,C). While this carina extends posteriorly past the metapleural gland orifice, in *Amblyopone* and many other ants in the clades Formicia and Poneria (i.e., the “formicoid” and “poneroid” clades), this carina extends to the ventral lip of the orifice, changing the orientation and shape of the orifice. The vertical subtending carina may also start from the ventrolateral metapectal carina (vlmtpc, Figures 2A,D and 4A,C) in other ants. The dorsal metapleural gland flange may be reduced in or absent in other Formicidae, and has phylogenetically informative variation, along with all other ornamentation surrounding the metapleural gland orifice and evaporatorium.

### 4.2 Apomorphies of the Mesosoma of *Protanilla*

In addition to variation outlined in the groundplan section above, the mesosoma of *P. lini* displays a number of apomorphies. The muscular configuration in *P. lini* is similar to that of *Formica* (Aibekova et al. 2022) and *Myrmecia* (Liu et al. 2019), yet the dorsal longitudinal cervical muscle (Idlm1) is conspicuously lacking, a rare condition among insects (e.g., Friedrich and Beutel 2008; Liu et al. 2019). Cervical muscles generally play an important functional role for ants when carrying objects with mouthparts, but the mechanical effect of the loss of ldlm1 is unclear. One of the muscles between the meso‐ and metafurca (IIvlm3) is also absent. Flight muscles are completely reduced, as in other ant workers previously examined (e.g., Liu et al. 2019; Aibekova et al. 2022), with the noteworthy exception of *Myrmica* (Janet 1898).

The pronotum of *Protanilla* is elongated as in *Martialis* (Rabeling et al. 2008: figures 1 and 2), a derived condition relative to the anteroposteriorly compact shape in *Formica* (Aibekova et al. 2022), the Myrmicinae, and other ants (Keller 2011: figures 21 and 22). It is conceivable that pronotal elongation enhances head mobility. Since variation in this feature is clinal, this character state is of minor phylogenetic significance. Although retention of flexibility in the promesonotal articulation is plesiomorphic, the degree of flexibility observed in *Protanilla* is enhanced, which we now see is due to expansion of the mesonotal glissella.

In contrast to the dome‐shaped, distinctly convex mesonotum observed in most ants (Keller 2011) the mesonotum of *Protanilla* is flat, as in *Martialis* (e.g., Rabeling et al. 2008: figures 1 and 2). The lone exception occurs in the *P. taylori* species‐group (*Protanilla helenae* [Borowiec et al. 2011]; Borowiec et al. 2011: figure 6). An arched mesonotum is also present in outgroup taxa capable of flight (e.g., Snodgrass [1956]) and is therefore a plesiomorphy for the Formicidae. The presumably derived conditions—a flat mesonotum, or one continuous with the metanotum and dorsal propodeum—have likely evolved multiple times in ants (Keller 2011: Appendix 2). Likewise, the rigid connection of both mesonotum and mesopleural region in *Martialis* (Brandão et al. 2010: figure 2B) and the Leptanillinae (Yamada et al. 2020: figure 6A; Griebenow 2024: figures 6A and 9A) has evolved independently in different lineages, for instance in *Oecophylla*, *Dorylus*, and *Tatuidris* (Keller 2011: Appendix 2). This derived condition increases compactness of the mesothorax relative to the ancestral condition (Aibekova et al. 2022: figure 1A; Keller 2011: Appendix 2; Liu et al. 2019) in which both areas are distinctly separated by a suture.

The metanotum of *Protanilla* is obliterated as in other Leptanillomorpha (Rabeling et al. 2008; Brandão et al. 2010: figure 2B; Yamada et al. 2020: figure 6A–C; Griebenow et al. 2022: figure 6A,B) and many ants beyond that clade (Boudinot, Khouri et al. 2022: figure 2; Aibekova et al. 2022: figure 2; Liu et al. 2019; Keller 2011). The metanotal region in *Protanilla* is laterally delimited by closed metathoracic spiracles and traversed by a series of longitudinal carinulae set in the metanotal groove; these carinulae are possibly an autapomorphy of *Protanilla* in the context of Formicidae. The metapleural region is largely fused with the propodeum but still separated from it by a longitudinal bulge of the metapleural gland bulla below the propodeal spiracle (Figures 1 and 2), a derived condition compared to *Formica*, where both posterior mesosomal regions are separated by an indistinct metapleural sulcus (Aibekova et al. 2022: figure 2B).

The legs of *Protanilla* differ from those of other ants (e.g., Aibekova et al. 2022: figure 13; Keller 2011: figure 24) mainly in the presence of paired medial setae on protarsomere 2 (Griebenow 2024: figure 25A), which is an autapomorphy of *Protanilla* among the Formicidae. Although the polarities of various pretarsal conditions need to be evaluated, it is worth noting that the arolia are well‐developed, although since *Protanilla* is subterranean, with rare epigaeic ventures (Griebenow 2020; Hamer et al. 2024), the necessity of a large arolium for moving efficiently on smooth surfaces (Beutel and Gorb 2006, 2008) is limited.

### 4.3 Comparison of *Protanilla* to Other Leptanillomorpha

No complex or unique derived features shared by workers of the Martialinae and Leptanillinae were found in the present study. A sister‐group relationship between *Martialis* and the Leptanillinae is well‐established (Romiguier et al. 2022; Borowiec et al. 2025; although see Cai 2024 and its rebuttal, Boudinot and Lieberman in press), with these together comprising the Leptanillomorpha *sensu* Richter et al. (2021). All leptanillomorphs are hypogaeic predators (e.g., Rabeling et al. 2008: figure 2; Richter et al. 2021), and some derived features of the clade may be correlated with living in upper soil layers or in subterranean tunnels. These include complete absence of worker eyes, with the single known exception of *Protanilla izanagi* Terayama (Griebenow 2024); possibly reduced pigmentation (Richter et al. 2021), although pigmentation is variable across the subfamily; and arguably also the simple setation observed in *Protanilla*, predominantly consisting of a regular vestiture of type 1 setae (c. 80 µm). It may be worthwhile to more formally study the evolution of these and other features to elucidate their relation to a hypogaeic lifestyle across Leptanillomorpha and other ants and to clarify in how far they can be considered synapomorphies of this clade.

Since the micro‐CT data published here are the first available for the worker mesosoma of any leptanillomorph, our capacity for detailed morphological comparison of the mesosoma of *P. lini* with other representatives of Leptanillomorpha is limited. SEM‐informed descriptions of mesosomal sclerites are available for *Martialis* (Brandão et al. 2010) and *Opamyrma* (Leptanillinae: Opamyrmini) (Yamada et al. 2020), allowing substantive comparison of the worker mesosoma in *P. lini* (and *Protanilla* by extension) to these two monotypic genera. No such studies are yet available for *Leptanilla*. Since the worker caste in two out of the five informal species‐groups of *Leptanilla* remains unknown (Griebenow 2024), caution is warranted in generalizing conditions in the worker mesosoma across that genus.

We can at least infer that the mesosoma of *Protanilla* shows overall plesiomorphic conditions relative to other described leptanillomorph genera. For instance, the strongly convex upper mesopleural region and the raised lower mesopleural area are separated by a distinct transverse mesopectal sulcus (Figure 2A), a symplesiomorphy of *Protanilla* relative to all other Leptanillomorpha, so far as is known (e.g., Brandão et al. 2010: figure 2B; Yamada et al. 2020: figure 6A; Griebenow 2024: figures 4A, 6A, and 9A). Total reduction of the metanotal groove and meso‐metapleural suture, which may be a corollary of miniaturization, is an apomorphy of *Opamyrma* (Yamada et al. 2020) and *Leptanilla* (Griebenow 2024) relative to *Protanilla*.

*Protanilla lini* and *Opamyrma* show plesiomorphic characters relative to *Martialis* (Brandão et al. 2010: figure 2B) in the presence of a distinct anterior mesonotal contact surface, which forms the contact with the pronotum, and the presence of a strongly pronounced U‐shaped epicnemial carina, which divides the mesothorax into anterior and posterior portions (Figure 2A; Yamada et al. 2020: figure 6D). Conversely, the condition of the mesonotal contact surface has never been comprehensively surveyed in *Leptanilla*, but the epicnemial carina is known to be absent in at least two members of the *Leptanilla thai* species‐group (Griebenow et al. 2025). At least these cases corroborate an overall tendency towards derivation in the mesosoma of *Leptanilla* relative to that of *Protanilla*.

## Author Contributions

**Lazzat Aibekova:** conceptualization, methodology, software, data curation, investigation, formal analysis, funding acquisition, writing – original draft. **Adrian Richter:** conceptualization, investigation, data curation, funding acquisition, writing – original draft. **Rolf G. Beutel:** conceptualization, investigation, project administration, supervision, writing – original draft. **Thomas van de Kamp:** data curation, methodology, writing – review and editing, software, resources. **Evan P. Economo:** funding acquisition, resources, supervision. **Zachary Griebenow:** conceptualization, investigation, validation, writing – original draft. **Brendon E. Boudinot:** conceptualization, data curation, funding acquisition, investigation, project administration, supervision, visualization, writing – original draft.

## Conflicts of Interest

The authors declare no conflicts of interest.

## Data Availability

The data that support the findings of this study are openly available in Zenodo at https://zendodo.org/, reference number doi: 10.5281/zenodo.14178456. The original DICOM files of the µ‐CT scan are available from Zenodo, doi: 10.5281/zenodo.14178456. The synchrotron data will be made available on request.
